# Frontotemporal Lobar Dementia Mutant Tau Impairs Axonal Transport through a Protein Phosphatase 1γ-Dependent Mechanism

**DOI:** 10.1523/JNEUROSCI.1914-20.2021

**Published:** 2021-11-10

**Authors:** Benjamin Combs, Kyle R. Christensen, Collin Richards, Andrew Kneynsberg, Rebecca L. Mueller, Sarah L. Morris, Gerardo A. Morfini, Scott T. Brady, Nicholas M. Kanaan

**Affiliations:** ^1^Department of Translational Neuroscience, Michigan State University, Grand Rapids, Michigan 49503; ^2^Neuroscience Program, Michigan State University, East Lansing, Michigan 48824; ^3^Hauenstein Neuroscience Center, Mercy Health Saint Mary's, Grand Rapids, Michigan 49503; ^4^Department of Anatomy and Cell Biology, University of Illinois at Chicago, Chicago, Illinois 60612; ^5^Marine Biological Laboratory, Woods Hole, Massachusetts 02543

**Keywords:** Alzheimer's disease, axonal transport, neurodegeneration, protein phosphatase 1, tau protein, tauopathies

## Abstract

Pathologic tau modifications are characteristic of Alzheimer's disease and related dementias, but mechanisms of tau toxicity continue to be debated. Inherited mutations in tau cause early onset frontotemporal lobar dementias (FTLD-tau) and are commonly used to model mechanisms of tau toxicity in tauopathies. Previous work in the isolated squid axoplasm model demonstrated that several pathogenic forms of tau inhibit axonal transport through a mechanism involving activation of protein phosphatase 1 (PP1). Here, we determined that P301L and R5L FTLD mutant tau proteins elicit a toxic effect on axonal transport as monomeric proteins. We evaluated interactions of wild-type or mutant tau with specific PP1 isoforms (α, β, and γ) to examine how the interaction contributes to this toxic effect using primary rat hippocampal neurons from both sexes. Pull-down and bioluminescence resonance energy transfer experiments revealed selective interactions of wild-type tau with PP1α and PP1γ isoforms, but not PP1β, which were significantly increased by the P301L tau mutation. The results from proximity ligation assays confirmed the interaction in primary hippocampal neurons. Moreover, expression of FTLD-linked mutant tau in these neurons enhanced levels of active PP1, also increasing the pausing frequency of fluorescently labeled vesicles in both anterograde and retrograde directions. Knockdown of PP1γ, but not PP1α, rescued the cargo-pausing effects of P301L and R5L tau, a result replicated by deleting a phosphatase-activating domain in the amino terminus of P301L tau. These findings support a model of tau toxicity involving aberrant activation of a specific PP1γ-dependent pathway that disrupts axonal transport in neurons.

**SIGNIFICANCE STATEMENT** Tau pathology is closely associated with neurodegeneration in Alzheimer's disease and other tauopathies, but the toxic mechanisms remain a debated topic. We previously proposed that pathologic tau forms induce dysfunction and degeneration through aberrant activation of a PP1-dependent pathway that disrupts axonal transport. Here, we show that tau directly interacts with specific PP1 isoforms, increasing levels of active PP1. Pathogenic tau mutations enhance this interaction, further increasing active PP1 levels and impairing axonal transport in isolated squid axoplasm and primary hippocampal neurons. Mutant-tau-mediated impairment of axonal transport was mediated by PP1γ and a phosphatase-activating domain located at the amino terminus of tau. This work has important implications for understanding and potentially mitigating tau-mediated neurotoxicity in tauopathies.

## Introduction

The microtubule-binding protein tau is a major component of the axonal cytoskeleton, and its normal physiological role remains a matter of debate (Guo et al., 2017). Pathologic tau modifications, including phosphorylation of specific residues and formation of multimers, are closely associated with Alzheimer's disease (AD) and related tauopathies. These modifications are typically linked to tau conformation changes that spatially and temporally correlate with dysfunction and dying-back degeneration of neurons within affected brain regions across different tauopathies (Kovacs, 2015; Kneynsberg et al., 2017). Most tauopathies are sporadic, but a number of inherited mutations within the *MAPT* (microtubule-associated protein tau) gene encoding tau induce early onset neurodegeneration classified within frontotemporal lobar dementias (FTLD-tau; Hutton et al., 1998; Ghetti et al., 2015; Forrest et al., 2018). Specific single-point mutations (e.g., the P301L and R5L mutations) can induce disease in humans and promote neurologic phenotypes in animal models of tauopathies (Lewis et al., 2000; Poorkaj et al., 2002; Tatebayashi et al., 2002; Santacruz et al., 2005).

Mutations alter conformation, phosphorylation state, microtubule binding, isoform composition, and/or aggregation propensity of tau protein in tauopathies (Ghetti et al., 2015). Any of these modifications could enhance tau toxicity in disease, but many questions remain about the underlying molecular mechanisms. Similarly, questions on whether pathologic modifications alter normal functions of tau, generate new biological activities, or both remain open. Much of the focus on the cellular functions of tau has centered on its affinity for microtubules and ability to promote microtubule assembly or stabilization *in vitro* (Weingarten et al., 1975). However, recent work identified functional roles of tau unrelated to regulation of microtubule dynamics. Notably, tau plays a role in signal transduction by regulating the localization and enzymatic activity of kinases and phosphatases involved in a diverse set of cellular processes (Lee et al., 1998; Liao et al., 1998; Ittner et al., 2010; Kanaan et al., 2013; Mueller et al., 2021).

Fast axonal transport (FAT), a complex set of microtubule-based intracellular trafficking events involving bidirectional movement of organelles along axons, is one cellular process affected by pathogenic tau. Using squid axoplasm preparations, a well-established experimental model to study FAT, we showed that physiological, or even supraphysiological, levels of normal human tau monomers did not negatively affect FAT (Morfini et al., 2007). In contrast, conformation-changing pathologic tau alterations, (aggregation/oligomerization or specific modifications to monomers), impaired FAT at physiological levels in this model (LaPointe et al., 2009; Kanaan et al., 2011; Patterson et al., 2011; Cox et al., 2016; Tiernan et al., 2016). This toxic effect was mediated through a protein phosphatase 1 (PP1)-dependent pathway that resulted in cargo dissociation from the major microtubule-based motor protein kinesin-1 and dependent on a biologically active motif in the extreme amino terminus of tau termed the phosphatase activating domain, (PAD; Morfini et al., 2004; LaPointe et al., 2009; Kanaan et al., 2011). Collectively, these findings indicated that pathologic tau modifications disrupt FAT by altering tau conformation and dysregulating signaling pathways through abnormal PP1 activation. Identification of putative PP1 binding sequences in tau suggested a direct interaction between it and PP1, but this was not directly assessed.

In this study, we test the hypothesis that two FTLD-tau mutations disrupt FAT via a PP1-dependent mechanism in mammalian neurons. Using protein–protein interaction assays we found that tau directly interacts with selected PP1 isoforms, and the disease-causing P301L mutation enhances such interactions. Measuring a regulatory phosphorylation site in PP1, we also show that tau increases active PP1 levels, an effect enhanced by FTLD-tau mutations. We demonstrate that both P301L and R5L mutant tau, but not wild-type (WT) tau, impair FAT using isolated squid axoplasm and cultured neurons as complementary experimental systems. In mammalian neurons, the tau mutants impaired FAT by increasing cargo pause frequency in a PP1γ isoform-dependent and PAD-dependent fashion. These findings establish a specific molecular mechanism by which two disease-causing tau forms similarly induce FAT impairments in mammalian neurons.

## Materials and Methods

### Recombinant protein purification

The tau constructs used here included full-length human tau (i.e., the hT40 or 2N4R isoform) in both WT- and FTLD-related P301L and R5L mutant forms (Hutton et al., 1998; Poorkaj et al., 2002). All tau cDNAs were tagged with C-terminal six-histidine (6xHis) tags and expressed in *Escherichia coli* using pT7C backbones and IPTG induction, as previously described (Combs et al., 2017). Briefly, recombinant proteins were separated using metal affinity chromatography controlled by an ÄKTA pure fast protein liquid chromatography machine (Cytiva) on a HiTrap Talon Crude column (Cytiva) followed by size-exclusion chromatography using a Hiprep 16/60 Sephacryl S-500 HR column (Cytiva). The samples were then further purified using a HiTrap Q HP anion-exchange column (Cytiva) using a salt gradient from 0 to 250 mm NaCl. DTT was added to a final concentration of 1 mm. All proteins were divided into single-use aliquots and frozen at −80°C. SDS-Lowry assays were used to determine protein concentrations (Combs et al., 2017).

### Vesicle motility assays in squid axoplasm

Isolated axoplasm from the squid *Doryteuthis pealeii* (Marine Biological Laboratory) was used to measure the effects of tau monomers on FAT, as previously described (Brady et al., 1993; LaPointe et al., 2009; Song et al., 2016). Monomeric WT, P301L, or R5L tau protein stocks were diluted in X/2 buffer containing the following (in mm): 175 potassium aspartate, 65 taurine, 35 betaine, 25 glycine, 10 HEPES, 6.5 MgCl_2_, 5 EGTA, 1.5 CaCl_2_, 0.5 glucose, and 5 adenosine triphosphate, pH 7.2, to a final concentration of 2 μm and perfused into isolated axoplasm. Anterograde and retrograde FAT rates were alternately obtained by matching the calibrated cursors to the bulk flow of membrane-bounded organelles (e.g., synaptic vesicles) for 50 min after perfusion, as described previously (Song et al., 2016). FAT rates over the last 20 min of the assay were compared between treatment groups as described previously (Cox et al., 2016; Tiernan et al., 2016).

### DNA cloning

DNA constructs were generated in a pCMV or pFIN backbone. The HaloTag (Promega) was attached to the N-terminal end of each of the PP1α, PP1β, and PP1γ1 isoforms (PP1γ exists as two alternatively spliced isoforms, but PP1γ1 is more prevalent in the brain and is the specific isoform used whenever PP1γ is referenced throughout this article; Strack et al., 1999). The NanoLuciferase tag (Promega) was attached to the C-terminal end of the WT, P301L, and R5L tau constructs. The P301L and R5L point mutations were generated using manufacturer instructions for the QuikChange Lightning Site-Directed Mutagenesis Kit (Agilent Technologies) in the pCMV vector and introduced into the pFIN vector via restriction enzyme cloning techniques.

### HaloTag-based pull-down assay

HEK293T cells (ATCC) were grown at 37°C and 5% CO_2_ in the following cell culture media: DMEM + L-glutamine (Invitrogen), 5% FBS, 1% penicillin/streptomycin. Cells were not used beyond passage 30.

Three hundred thousand HEK293T cells/well were plated into a 12-well polystyrene plate. After 24 h, the cells were transfected with 500 ng each of pCMV tau-NanoLuc (WT, P301L, or R5L) and pCMV Halo-PP1 (PP1α, PP1β, or PP1γ) or a pCMV Halo control (the HaloTag protein only) using Lipofectamine 2000 and allowed to express proteins for 18 h before collection. Immediately before cell lysis, 50 μl of HaloLink resin (25% slurry in 25% ethanol, Promega) was aliquoted for each sample. The beads were washed 3× in 500 μl of wash buffer (TBS, 0.05% NP-40, Sigma-Aldrich) with centrifugation steps of 800 × *g* for 2 min to settle resin between each wash. Cells were lysed in 150 μl of lysis buffer [50 mm Tris-HCl, pH 7.5, 150 mm NaCl, 1% Triton X-100, pepstatin (10 μg/ml), bestatin (10 μg/ml), leupeptin (10 μg/ml), phenylmethylsulfonyl fluoride (1 mm), aprotonin (10 μg/ml)], then homogenized 20× with disposable polypropylene dounce homogenization pestles (Sigma-Aldrich). The samples were centrifuged at 14,000 × *g* for 5 min at 4°C, and the supernatant was transferred to a new tube containing 350 μl of TBS (50 mm Tris, 150 mm NaCl, pH 7.4). Next, 450 μl of each sample lysate/TBS mix was transferred to the HaloLink resin beads, and the remainder of the lysates were frozen as preincubation lysate samples. The samples were incubated for 1 h on a rotating mixer to allow covalent binding of the HaloTagged PP1 or HaloTag-only as control. After incubation, samples were centrifuged at 800 × *g* for 2 min, and the supernatants were saved as post-pull-down lysate samples. The beads were washed 4× in 800 μl of wash buffer with the final wash step including a 5 min incubation on the rotating mixer. After removing the wash buffer, the beads were incubated in 50 µl of 2× sample buffer (40 mm Tris, pH 6.8, 4% SDS, 12% glycerol, 2% β-mercaptoethanol, 0.004% bromophenol blue) for 30 min at 1000 rpm and 30°C in an Eppendorf ThermoMixer C. After a centrifugation at 800 × *g* for 2 min, the supernatants were collected as the pull-down sample.

For immunoblots, 12.5 μl of preincubation and postincubation lysate samples were added to 2.5 μl of 6× sample buffer (final 1× composition was 20 mm Tris, pH 6.8, 2% SDS, 6% glycerol, 1% β-mercaptoethanol, 0.002% bromophenol blue). These samples and the pull-down samples (eluted in 2× sample buffer) were heated at 99°C for 5 min. Fifteen microliters of each of the lysate samples and 25 μl of the pull-down samples were separated using Criterion TGX Precast 4–20% gels (Bio-Rad) in SDS-PAGE. The proteins were then transferred to BioTrace NT nitrocellulose transfer membrane (Pall) at 400 mA for 50 min. The membranes were blocked in 2% milk/TBS for 1 h at room temperature and then probed overnight at 4°C with primary antibodies. All antibodies were diluted in 2% milk/TBS. The R1 polyclonal rabbit antibody (10 ng/ml; catalog #R1, Nicholas M. Kanaan at Michigan State University; RRID:AB_2832929) was used to detect tau (Berry et al., 2004). An anti-HaloTag mouse monoclonal antibody (1 µg/ml; catalog #G9211, Promega; RRID:AB_2688011) was used to detect HaloTag proteins. Blots were rinsed with 0.1% Tween 20/TBS (TBST) and then incubated with appropriate secondary antibodies diluted 1:20,000 in milk/TBS (IRDye 680RD goat anti-mouse IgG, catalog #926-68070, Li-Cor Biosciences; RRID:AB_10956588; and IRDye 800CW goat anti-rabbit IgG, catalog #926-32211, Li-Cor Biosciences; RRID:AB_621843). The blots were imaged using a Li-Cor Odyssey infrared imaging system, and Li-Cor ImageStudioLite 5.2 software was used to quantify immunoreactivity of bands. The bulk of Halo-PP1 or Halo tag alone covalently binds to the HaloLink resin and does not elute from the beads. Therefore, relative tau levels in pull-down samples were calculated by normalizing tau signal from the elution fraction to the change in Halo signal [tau elution signal/(Halo pre-pulldown signal – Halo post-pull-down signal)].

### Nano bioluminescence resonance energy transfer donor saturation assay

The nano bioluminescence resonance energy transfer (nBRET) technique using a donor and acceptor tags was used to demonstrate protein–protein interaction (Machleidt et al., 2015). The DNA constructs were expressed in low-passage HEK293T cells through Lipofectamine2000 (Thermo Fisher Scientific) transfection in a 12-well polystyrene plate (Corning). A donor saturation assay was used to demonstrate a specific interaction between the two proteins and to indicate the relative strengths of those interactions among the various tau and PP1 constructs. Each well was transfected with 10 ng of donor DNA (tau-NanoLuciferase) as well as an amount of acceptor DNA (Halo-PP1 or HaloTag-only control) ranging from 1000 to 1.4 ng for final acceptor:donor DNA ratios of 100:1, 33.3:1, 11.1:1, 3.7:1, 1.2:1, 0.4:1, and 0.1:1. A control well that was transfected with donor DNA (i.e., tau-luciferase) but no acceptor DNA (i.e., Halo-PP1 or Halo only) was included. An empty nonexpressing pTRE3G plasmid (Promega) was added to a final DNA concentration of ∼1 μg/transfection to act as a carrier DNA and standardize the amount of total DNA being transfected.

After 18 h, the cells were detached from the plate on addition of 500 μl of 0.5% trypsin and a brief incubation at 37°C. After dissociation, the trypsin was quenched with 1 ml of cell culture media. The cells were centrifuged at 200 × *g* for 2 min and resuspended in 1 ml of OptiMEM (no phenol red, 4% FBS, Invitrogen). Aliquots of each sample were diluted in trypan blue (Bio-Rad) for cell counting. The cells were diluted to a volume of 660 μl at 200 cells/μl in OptiMEM (no phenol red, 4% FBS). The cells were split into two samples. One received 0.33 μl of 618 ligand (Promega), and the other received 0.33 μl of DMSO as a control. The 618 ligand attaches to the HaloTag and acts as the acceptor fluorophore, whereas the DMSO control contains no fluorophore. Six wells of each transfection condition were plated onto a 96-well, white-wall, clear-bottom plate (three with 618 ligand and three with DMSO; Corning) at 20,000 cells and 100 μl/well then incubated for 18–20 h. A 5× stock solution of Nano-Glo substrate (Promega) was prepared in OptiMEM (no phenol red, 4% FBS). Twenty-five microliters of the Nano-Glo substrate stock was then added to each well and immediately placed in the BioTek Synergy NEO HTS plate reader equipped with filtered luminescence (BioTek). After shaking for 30 s, the luminescence values were read using a 410/80 bandpass filter (donor signal) and 610 nm long-pass filter (acceptor signal).

The mean corrected nBRET ratios were calculated according to the provided manufacturer protocol. The raw milliBRET unit (mBU) ratio was calculated by dividing the acceptor luminescence values by the donor luminescence values and then multiplying by 1000. These values were then corrected by subtracting the control milliBRET ratios (with DMSO) from the experimental milliBRET ratios (with 618 ligand). The mean and SD of these values were plotted versus the transfection DNA ratios and fit to a hyperbolic curve using GraphPad Prism.

### Primary neuron cultures

Timed-pregnant Sprague Dawley rats (Harlan Laboratories) were used to obtain embryonic d 18 (E18) fetuses (both sexes) for culturing primary hippocampal neurons, as previously described (Grabinski et al., 2015). Rats were housed in a room with a 12 h light/dark cycle and provided food and water *ad libitum*. Procedures were conducted in compliance with federal, state, and institutional guidelines and approved by the Michigan State University Institutional Animal Care and Use Committee.

Briefly, hippocampi were dissected from the rat embryos, cut into small pieces, and placed in ice-cold calcium- and magnesium-free buffer [CMF; Dulbecco's PBS (Invitrogen) with 0.1% glucose (Sigma-Aldrich), 2.5 μg/ml fungizone (Invitrogen), and 50 mg/ml gentamicin (Invitrogen)]. The tissue was washed 4× with CMF and incubated in 0.125% trypsin for 15 min at 37°C and washed again 2× with CMF then suspended in trypsin-inactivation solution (HBSS, Invitrogen), 50 μg/ml DNase I (Worthington), and 20% newborn calf serum (Invitrogen). Cells were dissociated through trituration using progressively smaller diameter syringes, pelleted over FBS (200 × *g* at 4°C) and then resuspended and plated in antibiotic-free neurobasal media (Invitrogen) supplemented with 1% GlutaMAX (Invitrogen) and 2% B-27 (Invitrogen).

### Lentivirus production

Lentiviral stocks were prepared to individually express WT tau, P301L tau, R5L tau, and GFP in primary neuron cultures. DNA constructs including a pHEF VSV-G envelope vector, a pNHP packaging vector (Coleman et al., 2003), and a pFIN construct to express the various versions of C-terminally FLAG-tagged tau under control of the chicken beta-actin promoter were cotransfected into four 150 mm culture dishes containing Hek293T cells at ∼50–60% confluency. A full cell culture media change was performed 2 h before transfection. The DNA was combined with 22.5 μg of pFIN tau, 15 μg of pNHP, and 7.5 μg of pHEF VSV-G DNA/plate, and then added to 2.4 ml of 0.1× TE (10 mm Tris, 1 mm EDTA, pH 8.0) and 1.2 ml sterile deionized water. Then 400 μl of 2.5 m CaCl_2_ was mixed into the solution and incubated for 5 min at RT followed by a dropwise addition of 4 ml of 2× HBS containing the following (in mm): 281 NaCl, 100 HEPES, and 1.5 Na_2_HPO_4_, pH 7.12, into the gently vortexed solution. The transfection solution was then evenly distributed throughout the plates at 2 ml/dish and incubated overnight. The media was then changed to viral cell culture media (DMEM, 2% FBS, 1% penicillin/streptomycin). The supernatants were collected on days 3 and 4 post-transfection by centrifuging at 675 × *g* for 5 min, then filtering through 0.45 μm filters. A 2 ml 20% sucrose bed was laid below the clarified media in ultracentrifuge tubes then spun in a swinging bucket rotor at 82,700 × *g* for 2 h at 4°C. The supernatant was removed, and the pellet was resuspended in 500 μl of PBS then aliquoted and frozen at −80°C after the second collection. Lentiviral titers were determined using a Lenti X P24 Rapid Titration Elisa Kit (TaKaRa Bio) according to manufacturer protocol, and similar levels of tau expression were confirmed via immunoblotting before use.

### Proximity ligation assay

Primary rat hippocampal neuron cultures were prepared as described above. Forty thousand cells were plated into each well of an eight-well chamber slide (Ibidi) coated with poly-D-lysine, and the cultures were treated with lentivirus at 0.25 pg of p24 protein/plated cell on day *in vitro* (DIV) 4 to express WT tau, P301L tau, or R5L tau. Additional control wells were left untreated with virus. After 4 d of incubation the cells were fixed with a 20 min treatment of 4% paraformaldehyde in cytoskeleton buffer containing the following (in mm): 10 MES, 138 KCl, 3 MgCl_2_, and 4 EGTA, pH 6.1, followed by three 5 min washes in TBS. The cells were permeabilized and incubated in blocking buffer (5% goat serum, 1% BSA, and 0.2% Triton X-100 in TBS) for 1 h at room temperature. Neurons expressing each tau construct and the untreated cells were then incubated with Tau7 antibody (catalog #Tau7, Nicholas M. Kanaan at Michigan State University; RRID:AB_2721195; Horowitz et al., 2006) at 2.5 ng/ml and a PP1 antibody (catalog #PA5-28218, Thermo Fisher Scientific; RRID:AB_2545694) or a PP1γ-specific antibody (catalog #07-1218, Millipore; RRID:AB_1977432) at 1:500 dilutions in 2% goat serum/TBS overnight at 4°C. Neurons expressing WT tau and untreated cells were incubated with each antibody individually or no primary antibodies for proximity ligation assays (PLAs) method controls. The Duolink PLA Control Kit (Sigma-Aldrich) was used to identify an association between tau and PP1 in the neurons. The cells were washed 3× 5 min at room temperature Buffer A then incubated with anti-mouse PLUS and anti-rabbit MINUS secondary probes (1:5 dilution each) in 2% goat serum for 1 h at 37°C. After 3 additional 5 min washes in buffer A, the neurons were incubated in ligase (1:40 dilution) in 1× ligation buffer for 30 min at 37°C and washed 3× in Buffer A again. Next, the neurons were incubated with polymerase (1:80 dilution) in 1× amplification buffer for 100 min at 37°C followed by incubation in blocking buffer for 1 h at room temperature. Then cells were incubated overnight at 4°C with biotinylated Tau12 antibody (catalog #Tau12, Nicholas M. Kanaan at Michigan State University; RRID:AB_2721192; Horowitz et al., 2006) at 2.5 ng/ml, and Tuj1, a β-III tubulin antibody (catalog #TuJ1, A. Frankfurter, University of Virginia, Department of Biology; RRID:AB_2315517; Caccamo et al., 1989) at 100 ng/ml in 2% goat serum. Tau12 is a human tau-specific antibody that does not label rodent tau and was used to identify human tau-expressing neurons, whereas Tuj1 will label all neurons. The cells were washed 3× 5 min with TBS then incubated with the following secondary antibodies: Streptavidin-conjugated Alexa Fluor 568 conjugate antibody (catalog #S11226, Thermo Fisher Scientific; RRID:AB_2315774) at a 1:1000 dilution and Alexa Fluor goat-anti mouse IgG2a 647 antibody (1:500; catalog #A32728, Thermo Fisher Scientific; RRID:AB_2633277) in 2% goat serum for 1 h at RT then washed again 3× 5 min in TBS.

The cells were imaged on a Nikon A1+ laser scanning confocal microscope equipped with 488, 561, and 640 solid state lasers using a 60× 1.40 numerical aperture (NA) objective and Nikon Elements AR software. We collected z-stack images over a depth of 2 µm with 0.25 µm steps (nine images/stack). The displayed images represent maximum intensity projections from the individual z-stack planes. Using a 40× 1.3 NA objective, we collected two to six images containing randomly selected, isolated neurons expressing exogenous tau (Tau12-positive) for each condition and replicate. Monochromatic images were produced for each channel, and the data were quantified using FIJI ImageJ. For the PLA puncta image (green channel) a threshold mask was generated using the default threshold setting, and the number of puncta per image was counted using the Count Particle function. The human tau signal (red channel) was identified using post-PLA immunocytofluorescence staining using the Tau12 antibody to label only exogenous tau as described above. A thresholding mask was generated using the Huang setting, and the total area of the mask was calculated using the Measure function and converted from pixels to nm^2^. The number of puncta/total area of Tau12 signal (nm^2^) was calculated for each image with a minimum cutoff of five pixels. The individual data points (see Fig. 4*C*) represent the mean of the results from all analyzed images within an individual replicate, and the experiment was repeated three independent times.

### Phospho-PP1 immunoblots

Low-passage HEK293T cells were plated into a poly-D-lysine-coated 24-well plate at 200,000 cells/well (experimental replications started with cells at the same initial passage number). After 24 h the cells were transfected with 0.5 µg of DNA. Polyethylenimine (PEI) was incubated at 55°C for 5 min before 5 µl was added to 50 µl of 150 mm NaCl for each well; 0.5 µg of DNA was also added to 50 µl of 150 mm NaCl. The PEI mix was added to the DNA mix, vortexed briefly, and incubated at room temperate for 20 min. The samples were then added to the wells in a dropwise manner. The lysates were collected in 75 µl of lysis buffer/well (20 mm Tris, 0.5 mm DTT, 300 mm NaCl, 0.5% Triton X-100, 2 μg/ml pepstatin, 2 μg/ml bestatin, 2 μg/ml leupeptin, 4 mm phenylmethylsulfonyl fluoride, 10 μg/ml aprotonin, 1 mm tetra-sodium pyrophosphate decahydrate, 10 mm β-glycerophosphate, 1 mm sodium orthovanadate, 1 m sodium fluoride, pH 7.5), and two wells for each condition were combined and sonicated with four pulses. Lysate protein concentrations were determined using the Bio-Rad protein assay as directed. Immunoblotting was performed as described above by loading 40 μg of total protein into 18-well gels that were transferred to nitrocellulose.

Primary rat hippocampal neurons (E18) were plated at 600,000 cells/well into six-well plates coated with poly-D-lysine as described above. On DIV 4 the neurons were treated with lentivirus (0.5 pg p24/cell) to induce expression of WT tau, P301L tau, R5L tau, or GFP. On DIV 8 lysates from individual wells were collected into 200 μl of the same lysis buffer described above and sonicated with four pulses. Cell lysate (55 μg of total protein) was loaded onto a 12-well gel for the PP1 blots and an 18-well gel (24 μg of total protein) for the tau and GFP blots, then transferred to nitrocellulose. The primary neuron blots were blocked in 2% milk-TBST for 1 h, washed 3× in TBST, then incubated with primary antibodies in 5% BSA-TBST overnight at 4°C.

Blots were probed for phospho-PP1 (pT320; 1:500; catalog #2581, Cell Signaling Technology; RRID:AB_330823), HaloTag (1:1000; catalog #G9211, Promega; RRID: AB_2688011), Total PP1 (E-9; 1:400; catalog #sc-7482, Santa Cruz Biotechnology; RRID:AB_628177), GAPDH (loading control; 1:2000; catalog #5174, Cell Signaling Technology; RRID:AB_10622025), Tau5 (10 ng/ml; catalog# Tau5, Nicholas M. Kanaan at Michigan State University; RRID:AB_2721194; LoPresti et al., 1995; Carmel et al., 1996), and GFP (1:30,000; catalog #ab290, Abcam; RRID:AB_303395). Blots were detected with secondary antibodies and imaged as described above. The pT320 PP1 (inactive form) signal was normalized to total PP1 (HaloTag signal for the Hek293T blots and total PP1 signal for the neuron blots) to obtain relative changes in the levels of active PP1. Notably, T320 in PP1α is analogous to T316 in PP1β and T311 in PP1γ. Tau signal was normalized to GAPDH signal to confirm similar levels of protein expression and loading.

### Live-cell FAT assay

Primary hippocampal neurons were prepared as described earlier and plated at 80,000 cells/well into four-well live cell chamber slides (Ibidi) coated with poly-D-lysine in 750 μl of neurobasal media (Invitrogen). The cells were transfected on DIV 8 using Lipofectamine 2000 reagent. A pFIN Synaptophysin-mApple and a pCMV plasmid containing one of the genes of interest (WT tau, P301L tau, R5L tau, Δ2–18 WT tau, Δ2–18 P301L tau, or GFP) were coincubated at 200 ng each in 30 µl of Opti-MEM serum-free media for 30 min. For each well, 0.5 µl of Lipofectamine2000 was also incubated in 30 µl of Opti-MEM media (Invitrogen) at room temperature for 30 min. The Lipofectamine mix was added to the DNA mix and incubated for another 30 min at room temperature. Sixty microliters of sample was added throughout the entirety of each well. After 2 h, half of the media (380 µl) was replaced with an equivalent volume of neurobasal media containing an antibody that binds the extracellular domain of neurofascin in the axon initial segment to differentiate the axon from dendrites during live cell imaging (250 ng/ml; catalog #75-172, Antibodies Incorporated; RRID:AB_2282826) as described previously (Hedstrom et al., 2008). At 24 h post-transfection, the media was replaced with neurobasal media containing Alexa Fluor goat-anti-mouse IgG2a 647 antibody (1 μg/ml; catalog #A32728, Thermo Fisher Scientific; RRID:AB_2633277).

After a 1 h incubation, the cells were imaged on the Nikon A1+ laser scanning confocal microscope using a 60× 1.40 NA objective and Nikon Elements AR software. The plates were maintained in a live cell chamber at 37°C and 5% CO_2_ throughout the imaging process. Imaging of transports was conducted in a main branch of the axon 50–150 µm distal from the axon initial segment. A region of 32 pixels by 128 pixels was bleached with 7 pulses of 1.7 s each from a 568 nm laser at 60% power. After 2 min a movie was generated through imaging at ∼28 frames per second (fps) for 5 min using the 568 nm laser to visualize movement of mApple-synaptophysin cargo. Kymographs were generated using the ImageJ macro KymoAnalyzer (Neumann et al., 2017). Tracks were drawn manually and subsequently analyzed by the macro to generate data. Anterograde and retrograde segment velocities represent a mean of each segment moving above a threshold of 0.3 µm/s. Each individual replicate in the primary neuron experiments represents the mean value from all analyzed kymographs within a given primary neuron preparation and represent completely independent culture runs from different pregnant females. For each condition, 1–4 neurons were analyzed per preparation with a minimum cutoff of 30 detectable tracks per kymograph (GFP: *n* = 6 independent experiments, 15 total neurons; WT tau: *n* = 6 independent experiments, 14 total neurons; P301L tau: *n* = 6 independent experiments, 15 total neurons; R5L: *n* = 5 independent experiments, 11 total neurons; see Fig. 7). All conditions included five independent replicates (WT tau, 9 total neurons; Δ2–18 WT tau, 8 neurons; P301L tau, 10 total neurons; Δ2–18 P301L tau, 9 neurons; see Fig. 10).

**Table 1.** Quantitative data for tau + shRNA axonal transport experiments

Experimental conditions	Anterograde segment velocity (µm/s)	Retrograde segment velocity (µm/s)	Total pause frequency (pauses/s)	Anterograde pause frequency (pauses/s)	Retrograde pause frequency (pauses/s)
Control shRNA					
WT tau	1.81 ± 0.33	1.93 ± 0.22	0.155 ± 0.038	0.072 ± 0.028	0.121 ± 0.025
P301L tau	2.00 ± 0.37	2.24 ± 0.27	0.356 ± 0.109	0.184 ± 0.055	0.206 ± 0.085
R5L tau	2.10 ± 0.34	2.32 ± 0.34	0.336 ± 0.112	0.206 ± 0.057	0.226 ± 0.086
PP1α shRNA					
WT tau	2.22 ± 0.36	2.26 ± 0.11	0.222 ± 0.039	0.158 ± 0.049	0.169 ± 0.025
P301L tau	2.30 ± 0.56	2.36 ± 0.27	0.339 ± 0.051	0.230 ± 0.063	0.237 ± 0.022
R5L tau	2.30 ± 0.20	2.24 ± 0.25	0.324 ± 0.059	0.253 ± 0.082	0.175 ± 0.040
PP1γ shRNA					
WT tau	1.85 ± 0.43	2.01 ± 0.29	0.168 ± 0.032	0.089 ± 0.019	0.126 ± 0.035
P301L tau	1.96 ± 0.30	2.08 ± 0.30	0.156 ± 0.020	0.087 ± 0.010	0.106 ± 0.015
R5L tau	2.00 ± 0.15	2.06 ± 0.21	0.227 ± 0.035	0.139 ± 0.046	0.155 ± 0.043

This table includes mean values ± SD for axonal transport directional segment velocities and total and directional pause frequencies from the tau expression groups within each shRNA treatment group (Fig. 7*E–I*).

**Table 2.** Two-way ANOVA Tukey's *post hoc* multiple comparison analysis for tau + shRNA axonal transport pause frequency data

Comparison	Total	Anterograde	Retrograde
Control shRNA			
WT tau versus P301L tau	*p* < 0.0001	*p* = 0.0100	*p* = 0.0109
WT tau versus R5L tau	*p* < 0.0001	*p* < 0.0001	*p* = 0.0014
P301L tau versus R5L tau	*p* = 0.8466	*p* = 0.7316	*p* = 0.7547
PP1α shRNA			
WT tau versus P301L tau	*p* = 0.0073	*p* = 0.0450	*p* = 0.0536
WT tau versus R5L tau	*p* = 0.0209	*p* = 0.0058	*p* = 0.9797
P301L tau versus R5L tau	*p* = 0.9163	*p* = 0.7061	*p* = 0.0821
PP1γ shRNA			
WT tau versus P301L tau	*p* = 0.9457	*p* = 0.9974	*p* = 0.7415
WT tau versus R5L tau	*p* = 0.2491	*p* = 0.2119	*p* = 0.5665
P301L tau versus R5L tau	*p* = 0.1406	*p* = 0.1875	*p* = 0.1939

This table includes the adjusted *p* values for the comparisons between tau expression groups within each shRNA treatment group for axonal transport total and directional pause frequencies. Data (Fig. 7*G–I*) was analyzed by two-way ANOVA with Tukey's multiple comparison test. Significance was established at an adjusted *p* ≤ 0.05.

### PP1 shRNA validation

We generated shRNA sequence pairs to achieve isoform-specific knockdown of PP1 as follows: PP1α: 5′-GATCCGACGATACAACATCAAACTTTCAAGAGAAGTTTGATGTTGTATCGTCTTTTTTA-3′ and 5′-AGCTTAAAAAAGACGATACAACATCAAACTTCTCTTGAAAGTTTGATGTTGTATCGTCG-3′; PP1γ: 5′-GATCCGCGGATATCGATAAACTCAATTCAAGAGATTGAGTTTATCGATATCCGTTTTTTA-3′ and 5′-AGCTTAAAAAACGGATATCGATAAACTCAATCTCTTGAATTGAGTTTATCGATATCCGCG-3′; and a nontargeting shRNA control: 5′-GATCCGTATAATACACCGCGCTACTTCAAGAGAGTAGCGCGGTGTATTATACTTTTTTA-3′ and 5′-TAAAAAAGTATAATACACCGCGCTACTCTCTTGAAGTAGCGCGGTGTATTATACGGATC-3′.

Knockdown of PP1 was evaluated using a Dual-Luciferase Reporter Assay System (Promega). HEK293T cells were plated into a poly-D-lysine-coated 24-well plate at 150,000 cells/well and transfected the next day. The transfection included 100 ng of a fusion construct containing *Renilla* luciferase and a rat cDNA sequence for one of the three PP1 isoforms in a psiCHECK-2 plasmid (also containing a separate firefly luciferase to control for expression); 400 ng of a pH1SH plasmid including shRNA sequences for PP1α, PP1γ, *Renilla* luciferase (positive control); a nontargeting control sequence (negative control); or empty for normalization. Two days after transfection, the cell culture media was replaced by 200 μl of Passive Lysis Buffer, and the cells were incubated on a shaker for 15 min. Then 20 μl of lysate was transferred to white opaque 96-well plates in triplicate and placed in a Promega GloMax-Multi Detection System. Following the Dual Luciferase two-injector protocol, the instrument injected 100 μl of Luciferase Assay Reagent II and measured the firefly luciferase activity. It then dispensed 100 μl of Stop & Glo Reagent and measured the *Renilla* luciferase activity. The *Renilla*:Firefly luciferase activity signal ratios were normalized to the average of the relevant *Renilla* fusion construct + empty pH1SH vector and reported as percent of control. This indicates the percentage knockdown of PP1 expression for each of the PP1 and control shRNA constructs.

We also confirmed mRNA knockdown in primary neurons using droplet digital PCR (ddPCR). Primary rat hippocampal neurons were plated at 150,000 cells/well into a 24-well plate and individually transduced on DIV four with lentiviruses to express shRNA targeting PP1α, PP1γ, or a scrambled shRNA control. RNA was extracted using a Qiagen RNeasy miniprep kit 8 d after transduction. The relative concentrations of PP1α and PP1γ mRNA were quantified using ddPCR. One nanogram of extracted RNA from each sample was diluted into a reaction mix as directed using the One-Step RT-ddPCR Advanced Kit for Probes (Bio-Rad). The reaction mix contained a final 1× Supermix concentration, 20 U/μl of reverse transcriptase, 15 mm DTT, 1× concentration of rat PP1α or PP1γ isoform-specific PCR primers and HEX fluorescent probe, and a 1× concentration of rat rplp0 PCR primers and FAM fluorescent probe as a reference gene. Oil droplets were generated using a QX200 automatic droplet generator (Bio-Rad), and 40 μl of droplets were transferred to a ddPCR 96-well plate (Bio-Rad), and the plate was covered with a foil cover and sealed. The reverse transcriptase reactions occurred at 50°C for 1 h in a C1000 Bio-Rad thermocycler. The PCR amplification protocol was 95°C for 10 min to activate the enzyme followed by 95°C for 30 s and 60°C for 60 s, repeated 40× with ramp rates of 3°C/s. The enzyme was deactivated at 98°C for 10 min and held at 4°C. A QX200 Droplet Reader (Bio-Rad) was used to quantify droplet fluorescence for each probe using the Absolute quantitation function of QuantaSoft software (Bio-Rad). The software calculated concentrations of PP1 and rplp0 RNA (copies/μl) and reported as a ratio of PP1 to the reference gene.

### Axonal transport with PP1 knockdown

The neurons were transfected with 175 ng of pCMV tau or GFP DNA, 175 ng of pH1SH shRNA DNA, and 100 ng of mApple-Synaptophysin DNA using Lipofectamine 2000, and the transport data collection and analysis was conducted using conditions identical to those described above. The number of independent experimental replicates and total number of neurons analyzed were as follows: WT tau + Control shRNA, *n* = 6, 11 neurons; WT tau + PP1α shRNA, *n* = 6, 9 neurons; WT tau + PP1γ shRNA, *n* = 6, 10 neurons; P301L tau + Control shRNA, *n* = 6, 12 neurons; P301L tau + PP1α shRNA, *n* = 6, 10 neurons; P301L tau + PP1γ shRNA, *n* = 6, 9 neurons; R5L tau + Control shRNA, *n* = 6, 10 neurons; R5L tau + PP1α shRNA, *n* = 6, 10 neurons; R5L tau + PP1γ shRNA, *n* = 6, 9 neurons.

### Experimental design and statistical analysis

**Figure 1.**
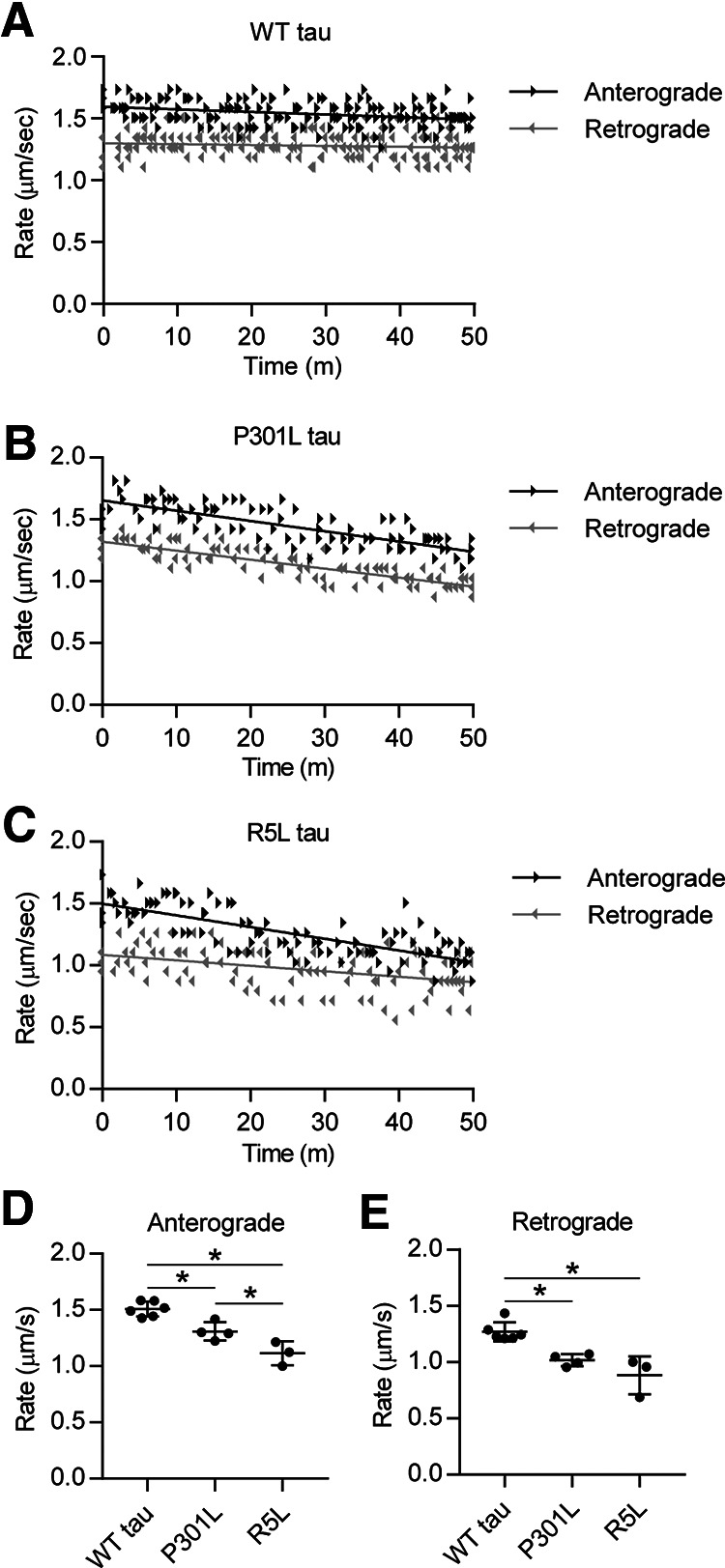
P301L and R5L tau disrupt fast axonal transport in squid axoplasm model. ***A***, Vesicle motility assays in the isolated squid axoplasm were used to measure the effects of tau proteins on FAT. Rate measurements (arrowheads, µm/s) from each independent experiment are plotted as a function of time (minutes). Black arrowheads represent anterograde FAT rates, and gray arrowheads represent retrograde FAT rates. Lines represent linear regression fits. WT tau monomer (2 μm, a near-physiological level) did not alter anterograde or retrograde FAT rates. ***B***, In contrast, perfusion of axoplasms with P301L tau monomers (2 μm) inhibited FAT rates in both directions. ***C***, Perfusion with R5L tau monomers at 2 μm also inhibited anterograde and retrograde FAT rates. ***D***, Quantitation of these effects revealed that compared with perfusion with WT tau (1.51 ± 0.07 μm/s), perfusion of P301L tau (1.31 ± 0.08 μm/s) or R5L tau (1.11 ± 0.11 μm/s) significantly decreased mean anterograde FAT rates. ***E***, Perfusion of P301L (1.02 ± 0.05 μm/s) and R5L tau (0.88 ± 0.17 μm/s) also decreased retrograde FAT rates compared with perfusion of WT tau (1.27 ± 0.09 μm/s). Statistical comparisons were made using a one-way ANOVA (WT tau, *n* = 6; P301L tau, *n* = 4; R5L tau, *n* = 3); data indicate mean ± SD; *p ≤ 0.05).

**Figure 2.**
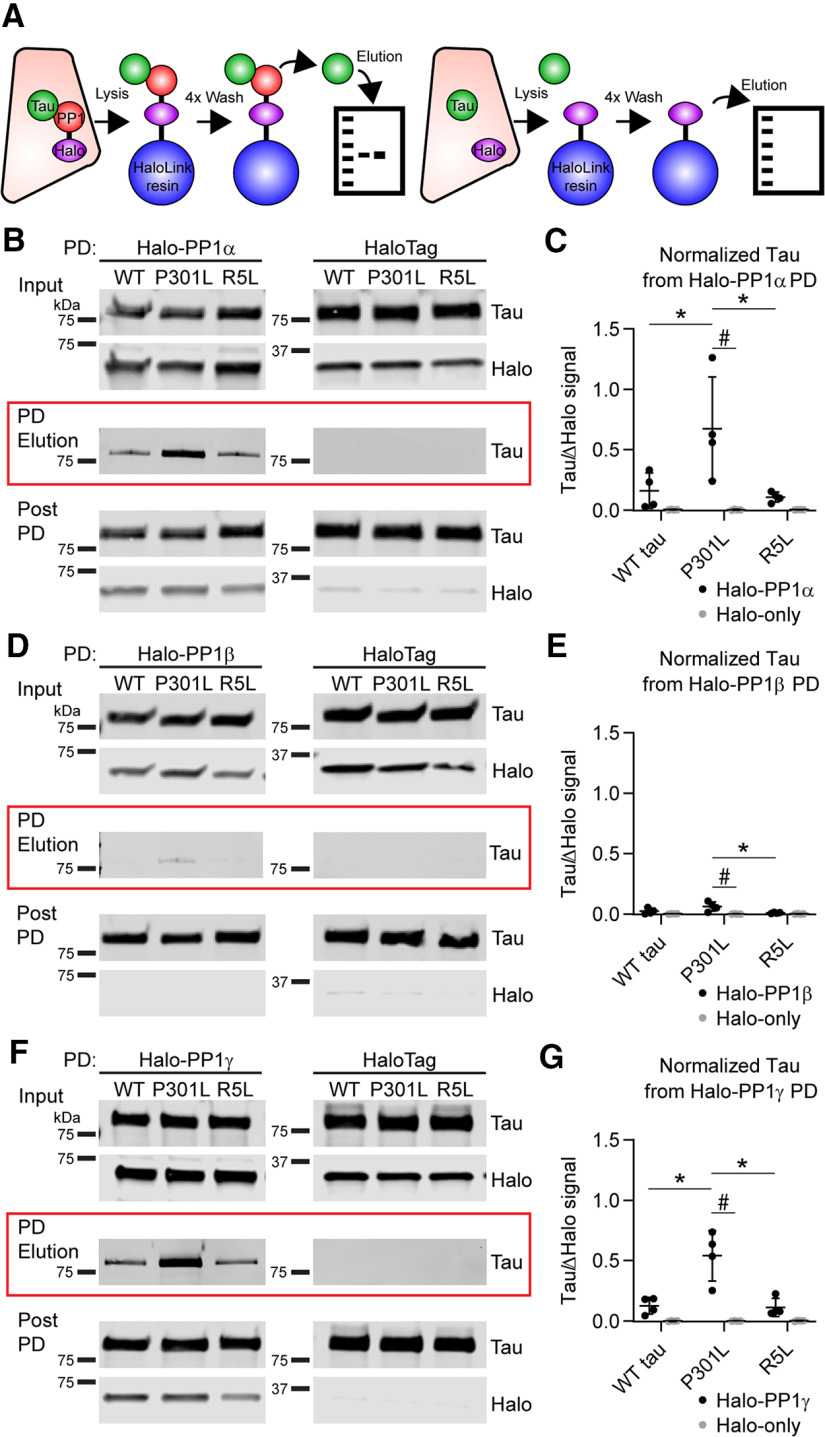
P301L mutation in tau enhances the interaction with PP1α and γ isoforms in pull-down (PD) assays. ***A***, Schematic showing assay design. Individual tau constructs were coexpressed with HaloTag-PP1 (left) or HaloTag-only controls (right) in HEK293T cells. Lysates prepared from these cells were incubated with HaloLink resin, which covalently binds to HaloTag. We quantified the amount of tau signal (pan-tau R1 antibody, green) eluted from each pull down by immunoblot (PD elution) and normalized to the reduction in Halo signal (anti-Halo antibody, red) from prebinding to postbinding lysate samples. ***B***, WT, P301L tau, and R5L tau proteins were detected in the elution samples after a pull down with Halo-PP1α (left, red box). Tau was not detected in the HaloTag-only control (right, red box). Lysate samples obtained before (Input) and after pull down with the HaloLink resin (Post PD) were probed to confirm similar protein expression and HaloTag binding to resin. ***C***, Quantitation of tau in the elution samples indicate that P301L tau (0.673 ± 0.429) significantly increases the interaction with PP1α when compared with WT (0.160 ± 0.147) and R5L tau (0.107 ± 0.041) and the control with HaloTag-only (WT tau, 0.004 ± 0.002; P301L, 0.003 ± 0.003; R5L, 0.003 ± 0.001). ***D***, A relatively low level of WT and P301L tau was eluted from the Halo-PP1β pull down but not the HaloTag-only control. The pre- and post-pull-down lysate samples from the Halo-PP1β and Halo-only lysates confirm similar expression and pull-down efficiency. ***E***, Quantitation of tau in the pull-down elution samples indicate that P301L tau (0.061 ± 0.038) significantly increases the interaction with PP1β when compared with the P301L + HaloTag control (−0.001 ± 0.001). Notably, the amount of tau in the elution with Halo-PP1β pull down is much lower for all forms (WT tau, 0.023 ± 0.024; R5L, 0.009 ± 0.005) and not significantly higher than the HaloTag-only controls (WT tau, 0.001 ± 0.001; R5L, −0.001 ± 0.001). ***F***, WT and P301L tau proteins were detected in the elution samples after a pull down with Halo-PP1γ. The pre- and post-pull-down lysate samples from the Halo-PP1γ and Halo-only lysates confirm similar expression and pull-down efficiency. ***G***, Quantitation of tau in the elution samples indicate that P301L tau (0.540 ± 0.209) significantly increases the interaction with PP1γ when compared with WT tau (0.125 ± 0.069), R5L tau (0.111 ± 0.075), and the P301L + HaloTag control (0.000 ± 0.001). Tau was also absent in HaloTag pull-down controls (WT tau, −0.001 ± 0.002; R5L, 0.001 ± 0.002). All data in the legend are reported as ratios of the signal intensities in arbitrary units. Statistical comparisons were performed using a repeated measures two-way ANOVA with Tukey's multiple comparison test, and each data point represents an independent experimental replicate (*n* = 4 independent replicates; data are mean ± SD; * *p* ≤ 0.05 for comparisons among the tau group; #*p* ≤ 0.05 for the comparison between Halo-PP1 and Halo-only pull downs).

**Figure 3.**
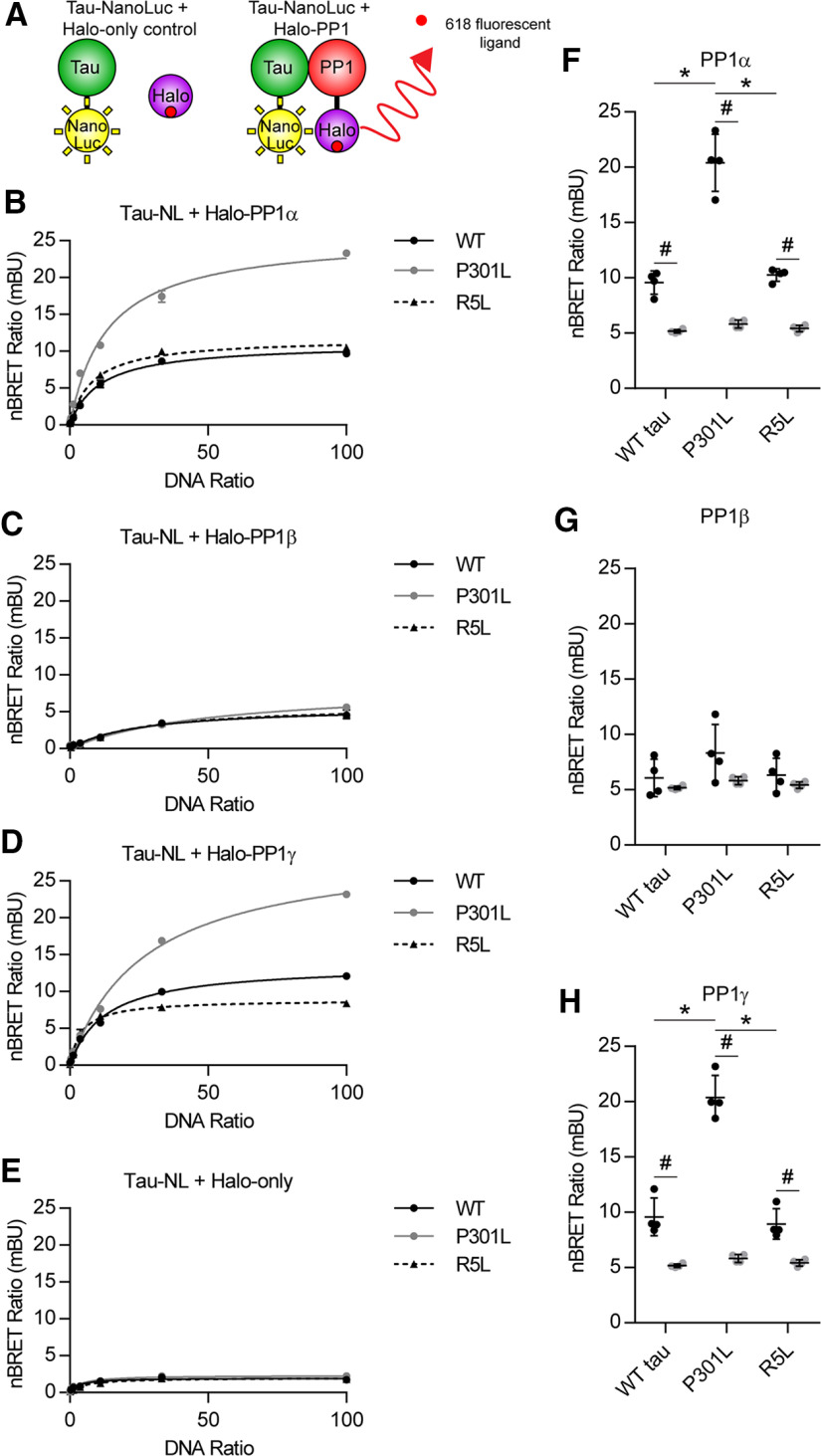
Tau protein interacts with PP1α and PP1γ, and the P301L tau mutation enhances the interaction in intracellular nBRET assay. ***A***, Tau proteins were tagged with a luciferase enzyme (NanoLuc) and coexpressed in HEK293T cells in the presence of an individual Halo-PP1 isoform or HaloTag-only control protein. A fluorescent ligand binds to the HaloTag, forming the acceptor molecule. Light emitted from the luciferase (donor) activates the fluorophore (acceptor) if they are within ∼37 Å of each other because of a specific protein–protein interaction. The donor and acceptor signals were measured using filtered luminescence and reported as the nBRET ratio. The acceptor:donor DNA ratio was varied to generate a donor saturation assay curve. ***B***, The hyperbolic shape of the donor saturation curves indicated a specific protein–protein interaction between Halo-PP1α and WT tau (solid black), P301L tau (solid gray), and R5L tau (dashed black) tagged with NanoLuciferase. ***C***, A strong interaction was not detected for the same forms of tau in the presence of Halo-PP1β. ***D***, Each form of tau displayed a clear interaction with Halo-PP1γ. ***E***, There was no evidence of a specific interaction between the Halo-only control and any of the tau proteins. ***F***, The nBRET was performed at an acceptor-to-donor DNA ratio of 100:1 with four independent replicates to compare nBRET ratios among the tau groups with Halo-PP1 or Halo-only. Each tau displayed significantly higher nBRET ratios with Halo-PP1α (WT tau, 9.57 ± 1.05 mBU; P301L, 20.40 ± 2.58 mBU; R5L, 10.25 ± 0.57 mBU) compared with Halo-only controls (WT tau, 5.168 ± 0.16 mBU; P301L, 5.814 ± 0.36 mBU; R5L, 5.418 ± 0.30 mBU). P301L tau nBRET ratios were significantly higher than WT and R5L tau with Halo-PP1α. ***G***, There was no significant increase detected for any of the individual tau constructs with Halo-PP1β (WT tau, 6.067 ± 1.69 mBU; P301L, 8.323 ± 2.59 mBU; R5L, 6.325 ± 1.53 mBU) compared with Halo-only or among the tau constructs and Halo-PP1β. ***H***, The nBRET ratios for each of the tau forms were significantly higher with Halo-PP1γ (WT tau, 9.579 ± 0.429 ± 1.71 mBU; P301L, 20.38 ± 1.99 mBU; R5L, 8.939 ± 1.37 mBU) compared with Halo-only controls. The nBRET ratios between Halo-PP1γ and P301L tau significantly increased compared with WT and R5L tau. Statistical comparisons for ***F–H*** were performed using a two-way ANOVA with Tukey's multiple comparison test, and each data point represents an independent experimental replicate (data are mean ± SD; *p ≤ 0.05 for the comparison among the tau groups; #*p* ≤ 0.05 for the comparison between Halo-PP1 and Halo-only groups).

**Figure 4.**
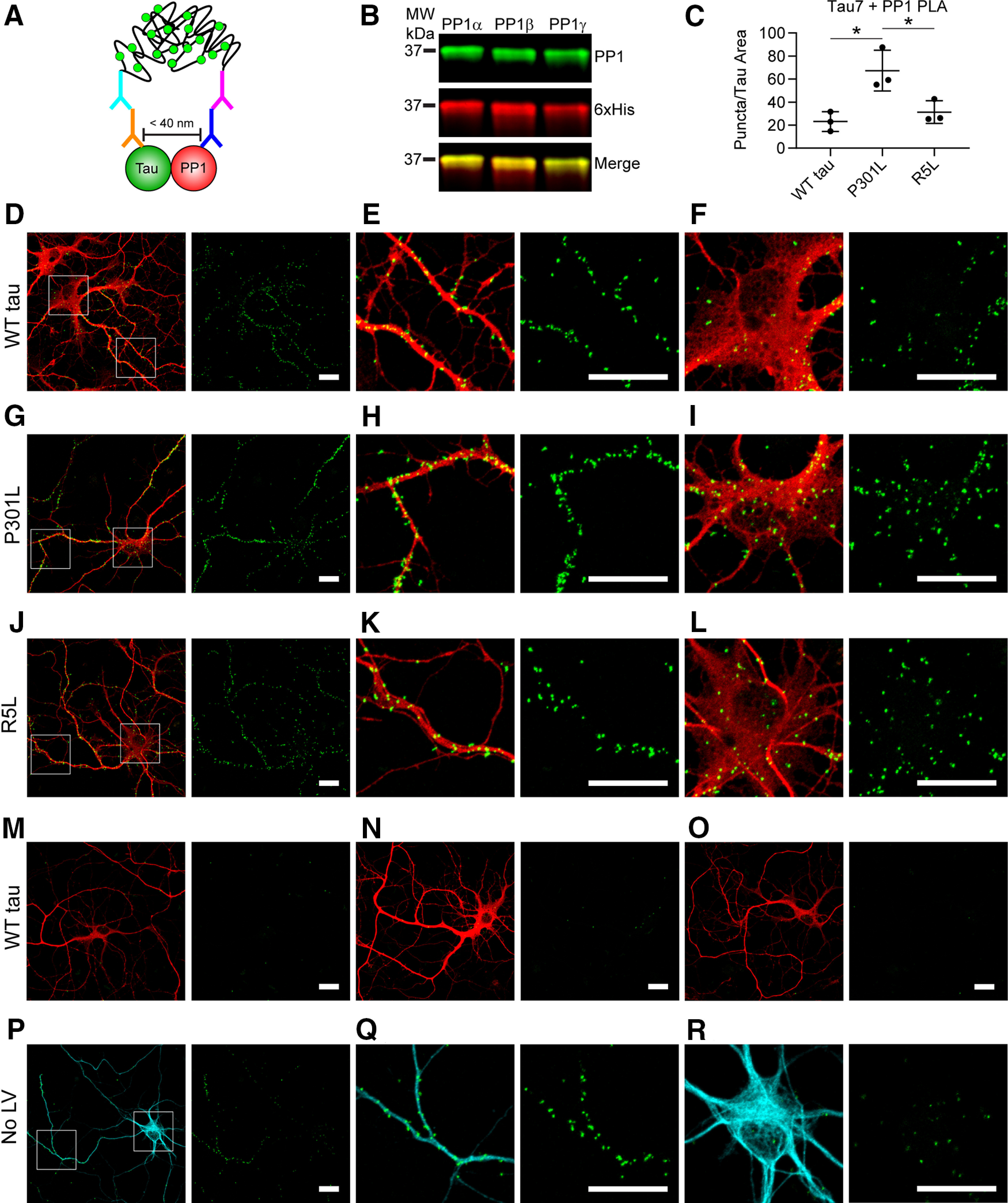
P301L tau increases the association with PP1 in primary hippocampal neurons. ***A***, Lentiviruses were used to express WT, P301L, or R5L tau in cultured primary rat hippocampal neurons beginning at DIV 4 and fixed in 4% paraformaldehyde on DIV 8. The neurons were probed with Tau7 (C-terminal tau epitope) and a PP1 antibody before performing the PLA staining procedure that demonstrates a close association between these proteins (<40 nm) indicated by a green fluorescent punctate signal. Following PLA, the cells underwent immunocytofluorescence staining with a Tau12 (red, human tau-specific N-terminal tau epitope) to identify exogenous tau and Tuj1 (cyan) to identify neurons and neuron processes. ***B***, The PP1 antibody recognizes recombinant PP1α, PP1β, and PP1γ in an immunoblot (green), and a 6× His antibody labels total protein. ***C***, Images of isolated neurons were used to quantify puncta per area of Tau12 staining (i.e., transduced neurons). P301L tau-expressing neurons (67.3 ± 17.7 puncta/nm^2^) showed significantly greater PLA signal than WT (23.1 ± 8.5 puncta/nm^2^) and R5L tau-expressing neurons (31.3 ± 9.8 puncta/nm^2^), complementing results obtained in HEK cell pull down and NanoBRET assays (Figs. 2, 3). Data represent mean ± SD and were analyzed using repeated measures one-way ANOVAs and Tukey's *post hoc* multiple comparison test, *p ≤ 0.05. ***D–F***, PLA detected associations between WT tau and endogenous rat PP1 (PLA, green puncta; Tau12, red), in both neuronal processes (***E***) and cell bodies (***F***). ***G–I***, Neurons expressing P301L tau displayed an enhanced association between tau and PP1 as evidenced by the PLA puncta, which were found in processes (***H***) and cell bodies of neurons (***I***). ***J–L***, Neurons expressing R5L tau showed an association between tau and PP1 that was similar to WT tau cells and localized to neuronal processes (***K***) and cell bodies (***L***). ***M–O***, Primary antibody delete controls included omission of either Tau7 (***M***), PP1 (***N***), or both (***O***). Primary delete controls showed little to no nonspecific background signal. ***P–R***, PLA in untransduced neurons (No LV) demonstrated an association between endogenous rat tau and PP1, which were evident in neuronal processes (***Q***) and cell bodies (***R***) of neurons. Scale bars: 20 μm for all images.

**Figure 5.**
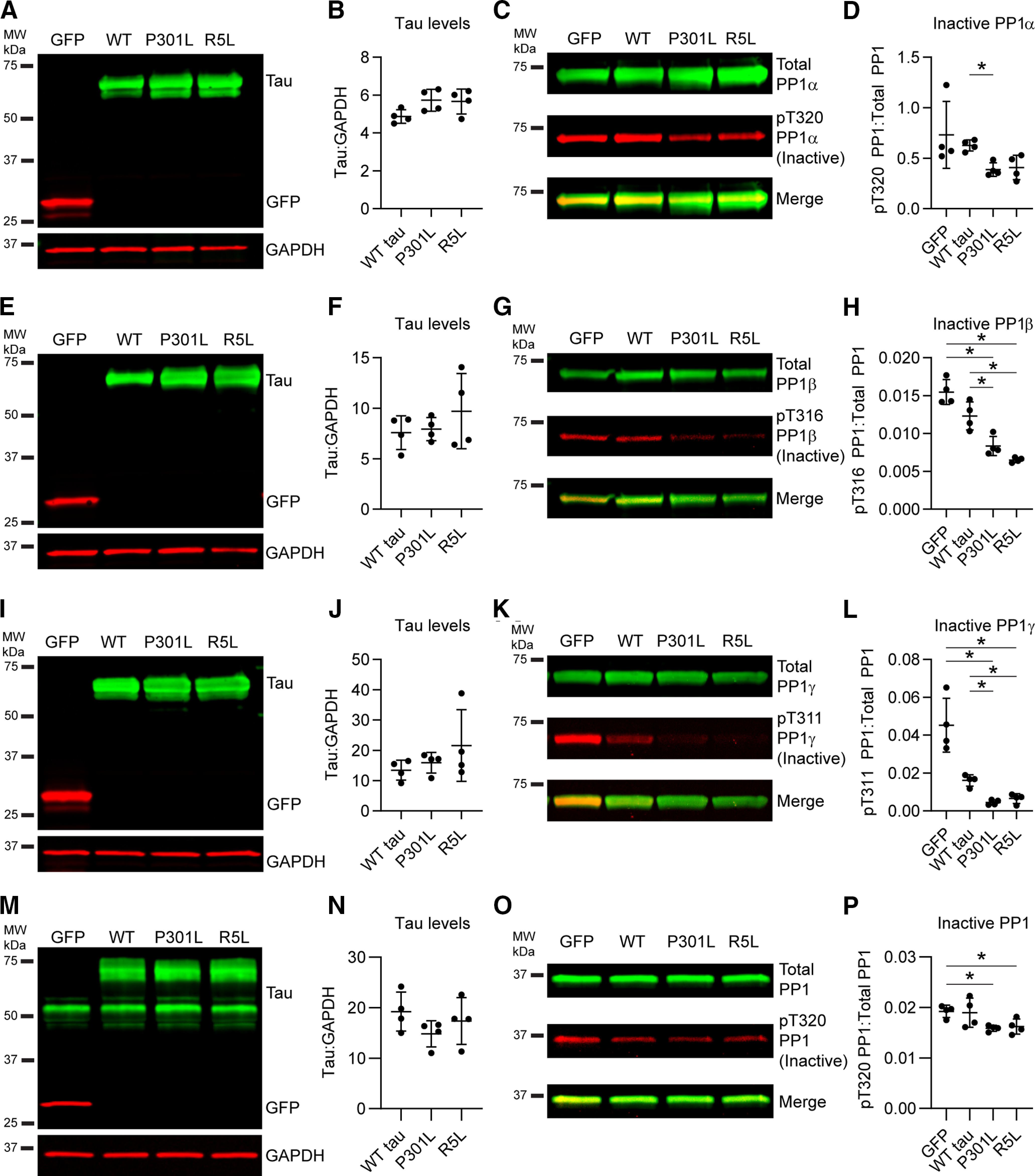
FTLD mutant tau enhances the levels of active PP1 in cells and primary neurons. ***A***, Immunoblotting confirmed expression of GFP, WT, P301L, and R5L tau in HEK293T cells coexpressing PP1α. ***B***, Quantification of tau bands indicated similar levels of tau across the three constructs (WT tau, 4.87 ± 0.36; P301L, 5.73 ± 0.58; R5L: 5.67 ± 0.66). ***C***, Immunoblots of total PP1 (green, HaloTag antibody) and inactive PP1 (red, phospho-Thr320 PP1 antibody). ***D***, Quantification of PP1 blots showed that P301L tau increased levels of active PP1α (as indicated by reduced inactive pT320 PP1 signal) compared with GFP control (GFP, 0.731 ± 0.331; WT tau, 0.627 ± 0.054; P301L, 0.388 ± 0.066; R5L, 0.409 ± 0.121). ***E***, Immunoblots of GFP and tau in cell lysates coexpressing PP1β. ***F***, Quantification of tau bands indicated similar expression of tau (WT tau, 7.58 ± 1.66; P301L, 7.94 ± 1.15; R5L, 9.72 ± 3.72). ***G***, Immunoblots of total PP1 (green) and inactive pT316 PP1β (red). ***H***, Quantification of PP1 blots showed that expression of any of the three forms of tau significantly increased active PP1β levels (i.e., reduced inactive pT316 PP1β) compared with GFP control (GFP, 0.0155 ± 0.0017; WT tau, 0.0123 ± 0.0019; P301L, 0.0084 ± 0.0013; R5L, 0.0065 ± 0.0003). P301L and R5L tau significantly increased active PP1β compared with WT tau as well. ***I***, Immunoblots of cells coexpressing GFP, WT tau, P301L tau, or R5L tau and PP1γ. ***J***, Quantification of tau bands showed similar tau expression levels (WT tau, 13.4 ± 3.3; P301L, 15.9 ± 3.4; R5L, 21.6 ± 11.8). ***K***, Immunoblots of total PP1 (green) and inactive pT311 PP1γ (red). ***L***, Quantification of PP1 blots demonstrated that expression of all forms of tau increased active PP1γ (i.e., reduced inactive pT311 PP1γ; GFP, 0.0453 ± 0.0142; WT tau, 0.0161 ± 0.0030; P301L, 0.0045 ± 0.0013; R5L, 0.0065 ± 0.0026). ***M***, Immunoblot of lysates from rat primary neurons expressing GFP or tau (WT, P301L, or R5L) via lentiviral transduction probed with tau (green) and GFP (red) and a loading control (GAPDH). ***N***, Quantification of the exogenous tau bands (top, green) indicated similar levels of tau across the three constructs (WT tau, 19.2 ± 3.9; P301L, 14.8 ± 2.6; R5L, 17.4 ± 4.6). ***O***, Immunoblot of total PP1 (green, PP1 antibody) and inactive PP1 (red, pThr320 PP1 antibody). ***P***, Quantification of the immunoblots demonstrated that P301L tau and R5L tau expression increased levels of active PP1 (i.e., reduced inactive pT320 PP1) compared with a control GFP expression, whereas WT tau did not significantly change active PP1 levels (GFP, 0.0192 ± 0.0012; WT tau, 0.0190 ± 0.0029; P301L, 0.0159 ± 0.0006; R5L, 0.0162 ± 0.0015). Note that tau signal was normalized to GAPDH loading control (***B***, ***F***, ***J***, ***N***), and pT320 PP1 signal was normalized to total PP1 signal (***D***, ***H***, ***L***, ***P***). Data in figure legend are reported in ratios of the signal intensities in arbitrary units. All data are mean ± SD and were compared using repeated measures one-way ANOVAs with a Geisser–Greenhouse correction and Tukey's *post hoc* multiple comparison test, *p ≤ 0.05).

**Figure 6.**
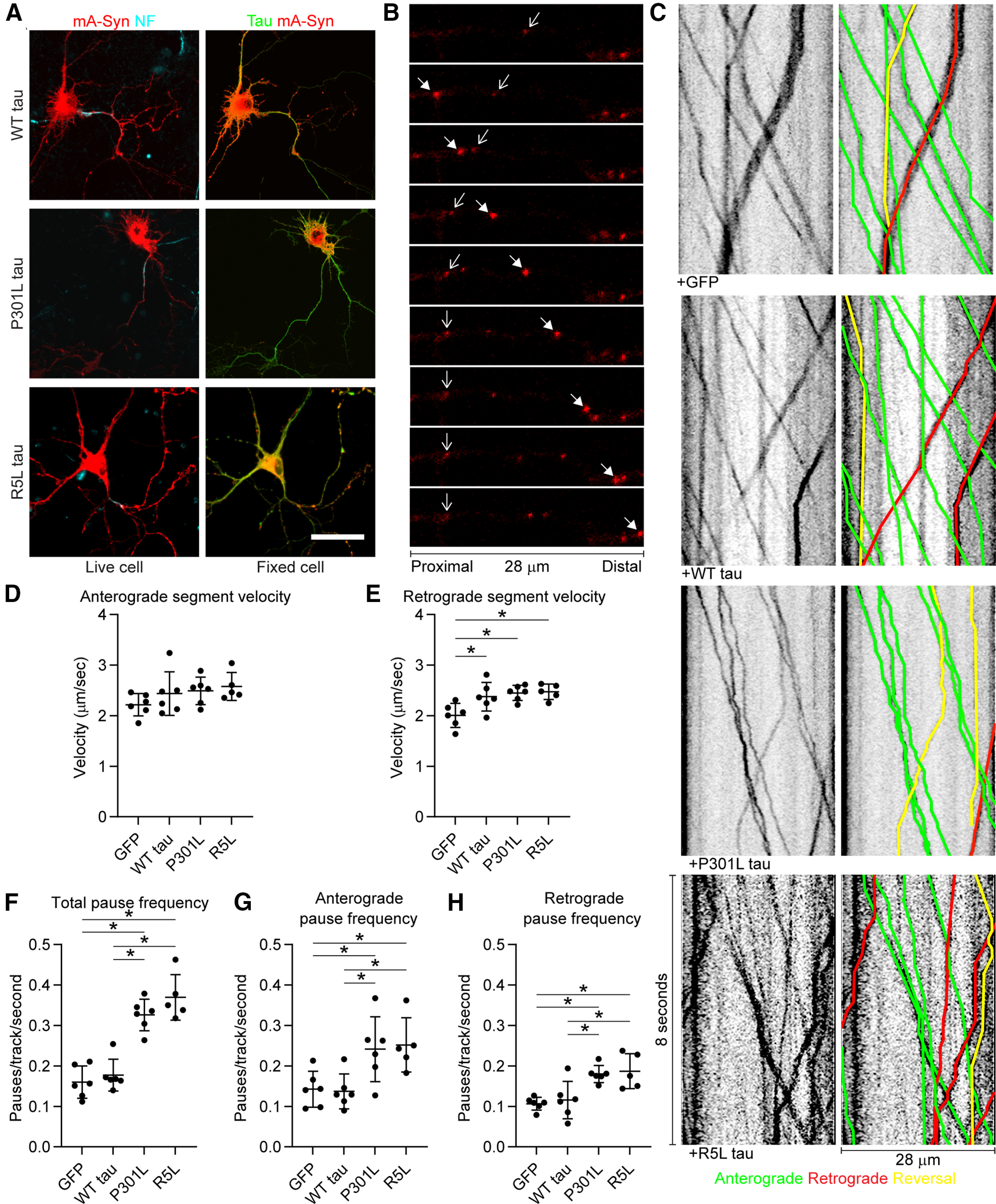
P301L and R5L tau increase axonal transport pause frequency in primary rat hippocampal neurons. ***A***, Neurons were cotransfected with an mApple-synaptophysin (mA-Syn) fusion construct and GFP, WT tau, P301L tau, or R5L tau. Axons were identified in live cells by labeling an external region of neurofascin (NF, left, cyan). After fixation cotransfection of both constructs was identified in all cells (right). Scale bar, 50 μm. ***B***, Axonal transport of fluorescent vesicles was imaged at 28 fps for 5 min. Each panel in this image is 1 s after the previous one. Scale bar, 28 μm. The solid arrows track a vesicle moving in the anterograde direction, and the wire arrows track a vesicle moving in retrograde direction before pausing. ***C***, Kymographs were generated and analyzed using the KymoAnalyzer macro for ImageJ. The parameters included direction, segment velocities, and pause frequency. Anterograde tracks are labeled green, retrograde tracks area labeled red, and tracks that switch direction are labeled yellow. ***D***, The mean anterograde segment velocity was not significantly affected by expression of GFP or any of the tau proteins tested (GFP, 2.22 ± 0.22 µm/s; WT tau, 2.44 ± 0.43 µm/s; P301L, 2.49 ± 0.27 µm/s; R5L, 2.58 ± 0.28 µm/s). ***E***, Expression of WT, P301L, or R5L tau caused a small but significant elevation in retrograde velocity (∼20%) when compared with GFP control but no significant differences were detected between the three tau proteins (GFP, 2.01 ± 0.24 µm/s; WT tau, 2.38 ± 0.28 µm/s; P301L, 2.45 ± 0.15 µm/s; R5L, 2.47 ± 0.15 µm/s). ***F***, Expression of either P301L or R5L tau resulted in an increase of total pause frequency (number of pauses/track/s) compared with GFP and WT tau expression (GFP, 0.160 ± 0.040 pauses/s; WT tau, 0.178 ± 0.039 pauses/s; P301L, 0.326 ± 0.039 pauses/s; R5L, 0.370 ± 0.057 pauses/s). ***G***, Anterograde pause frequency (pauses in cargo traveling in the anterograde direction) increased with P301L and R5L tau expression compared with GFP and WT tau (GFP, 0.143 ± 0.044 pauses/s; WT tau, 0.137 ± 0.043 pauses/s; P301L, 0.241 ± 0.080 pauses/s; R5L, 0.252 ± 0.067 pauses/s). ***H***, The retrograde pause frequency also increased on P301L and R5L tau expression compared with GFP and WT tau (GFP, 0.107 ± 0.015 pauses/s; WT tau, 0.116 ± 0.046 pauses/s; P301L, 0.180 ± 0.021 pauses/s; R5L, 0.187 ± 0.043 pauses/s). The groups were compared using a one-way ANOVA with Tukey's multiple comparison test, and each data point represents an independent replicate (*n* = 6 for GFP, WT tau, and P301L tau and *n* = 5 for R5L tau; the data are the mean ± SD; *p ≤ 0.05).

**Figure 7.**
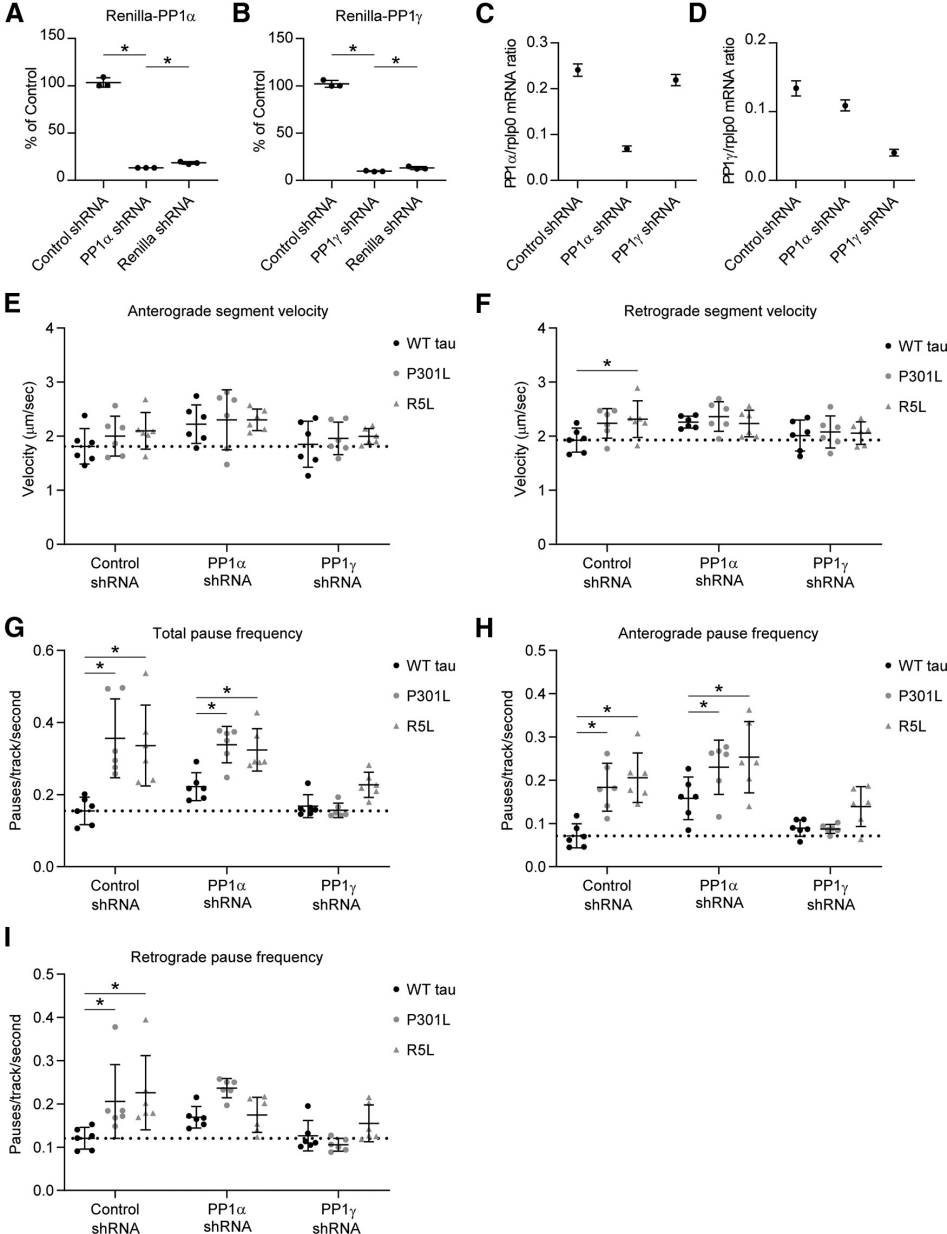
PP1γ knockdown rescues the effects of P301L and R5L on fast axonal transport pause frequency in primary neurons. PP1 expression was knocked down using isoform-specific shRNAs. The shRNAs were validated using a *Renilla*-Firefly Luciferase assay in HEK293T cells. *Renilla*-PP1 fusion constructs were cotransfected with shRNAs targeting Renilla (positive control), a nontargeting shRNA sequence (Control shRNA; used as a negative control), or specific PP1 isoforms. Expression knockdown is reported as percentage of an empty shRNA vector control. ***A***, PP1α shRNA produced 87% knockdown from control levels. ***B***, PP1γ shRNA induced 90% knockdown from control levels. Data were compared (***A–B***) using a one-way ANOVA with Tukey's multiple comparison test, and each data point represents an independent replicate (*n* = 3; data are mean ± SD; *p ≤ 0.05). ***C***, Neuronal mRNA knockdown was validated using lentiviral expression of PP1α-shRNA, PP1γ-shRNA, or the control shRNA. PP1α and PP1γ mRNA concentrations were determined using ddPCR and normalized to *rplp0* reference gene expression. PP1α shRNA treatment knocked down PP1α mRNA compared with control shRNA treatment, whereas PP1γ shRNA treatment only minimally reduced it. ***D***, PP1γ shRNA knocked down PP1γ mRNA compared with control shRNA treatment, whereas PP1α shRNA slightly reduced it. Error bars in ***C*** and ***D*** represent the 95% confidence intervals. ***E***, Transport experiments were repeated with PP1α, PP1γ-, or control shRNA. Anterograde segment velocity was not significantly different among any of the conditions. Dotted lines represent the baseline values (i.e., the mean of values in the WT tau + control shRNA treatment condition for ***E*** and ***F***. ***F***, Retrograde segment velocity was slightly higher with R5L expression compared with WT tau with control shRNA. ***G***, Expressing P301L tau or R5L tau increased total pause frequency compared with WT tau when treated with control or PP1α-shRNA. In contrast, PP1γ-shRNA treatment rescued P301L- and R5L-induced increases in pause frequency by reducing total pause frequency to WT tau control levels. ***H***, P301L and R5L tau increased anterograde pause frequency compared with WT tau + control or PP1α-shRNA. Treatment with PP1γ-shRNA reduced anterograde pause frequency in P301L and R5L neurons compared with WT tau neurons. ***I***, P301L and R5L expression increased retrograde pause frequency compared with WT tau with control shRNA treatments. Coexpression of WT, P301L, or R5L tau with PP1α-shRNA were not different likely because of the enhanced pause frequency in WT. With P301L and R5L, PP1γ-shRNA reduced retrograde pause frequency to levels similar to WT with PP1γ-shRNA and to baseline levels (i.e., WT with control shRNA). Data were compared (***E–I***) using a two-way ANOVA with Tukey's multiple comparison test to compare the three tau groups within each individual shRNA treatment group, and each data point represents an independent replicate (*n* = 6; the data are mean ± SD; *p ≤ 0.05; Table 1, mean ± SD; Table 2, for statistical *p* values).

**Figure 8.**
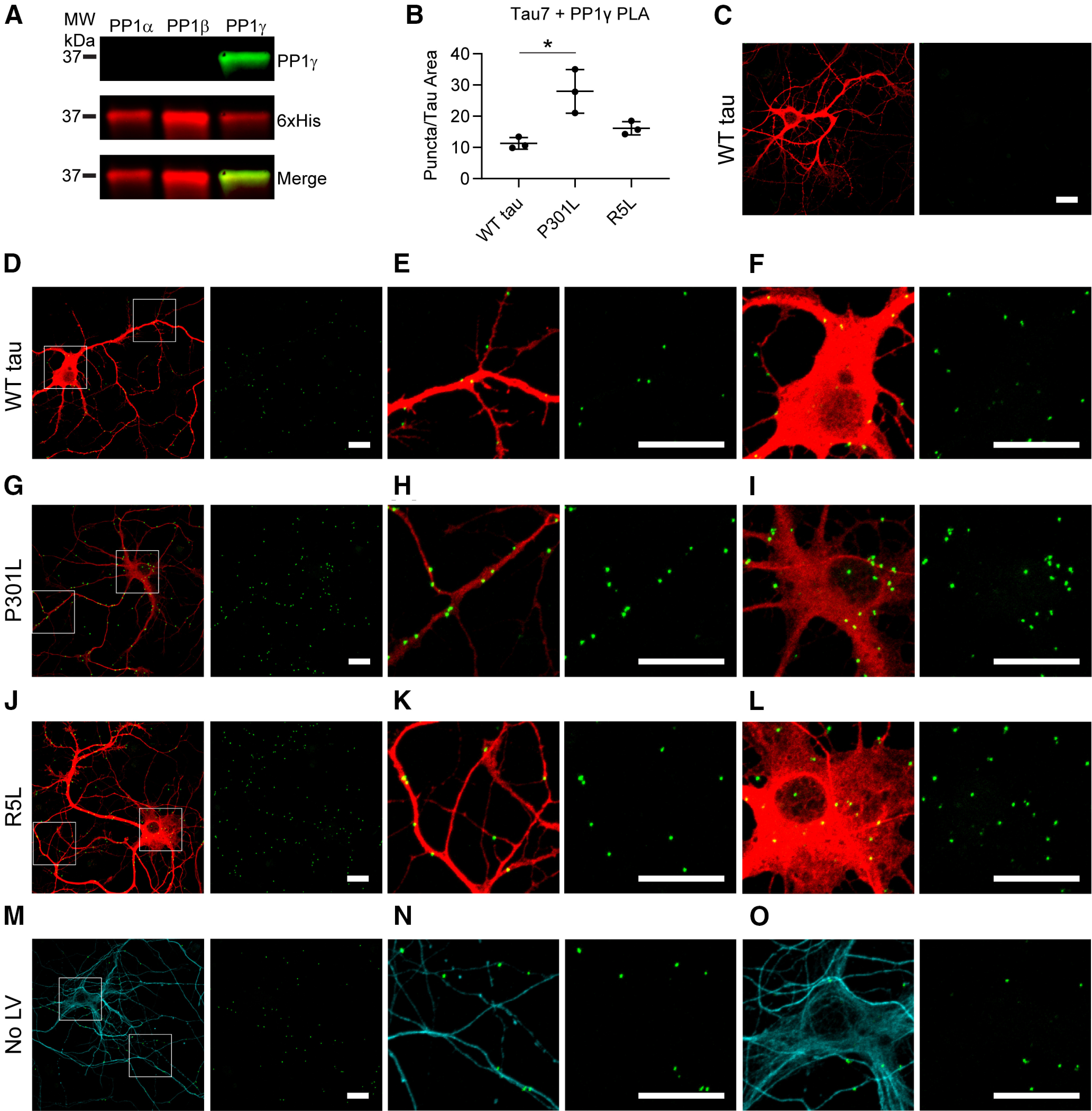
P301L tau increases the association with PP1γ isoform in primary hippocampal neurons. ***A***, PLA experiments were repeated with the Tau7 antibody and a PP1γ-specific antibody verified with immunoblot. Recombinant PP1 isoforms were probed with a PP1γ antibody (green) and a 6xHis antibody (red). ***B***, PLA puncta were quantified and normalized to area of Tau12 staining (i.e., exogenous tau staining). P301L tau (28.0 ± 7.0 puncta/nm^2^) expression increased levels of PLA signal compared with WT tau (11.3 ± 1.9 puncta/nm^2^), whereas R5L (16.2 ± 2.1 puncta/nm^2^) expression did not significantly change. Data represent mean ± SD of three replicates and were analyzed using a repeated measures one-way ANOVA and Tukey's *post hoc* multiple comparison test; *p ≤ 0.05. ***C***, A Tau7 primary antibody deletion demonstrated the lack of background signal when PLA is performed with the PP1γ antibody only. ***D–F***, Associations between WT tau and endogenous rat PP1γ (PLA, green puncta; Tau12, red) were identified in the neuronal processes (***E***) and cell bodies (***F***) of the neurons. ***G*–*I***, Lentiviral expression of P301L tau increased PLA puncta, which were found in processes (***H***) and cell bodies of neurons (***I***). ***J–L***, PLA in the presence of R5L tau also demonstrated an association between tau and PP1γ, localized to the processes (***K***) and cell bodies (***L***) of the neurons. ***M–O***, PLA was performed in untransduced neurons to identify an association between endogenous rat tau and PP1γ, also found in neuronal processes (***N***) and cell bodies (***O***). Scale bars: 20 μm for all images.

**Figure 9.**
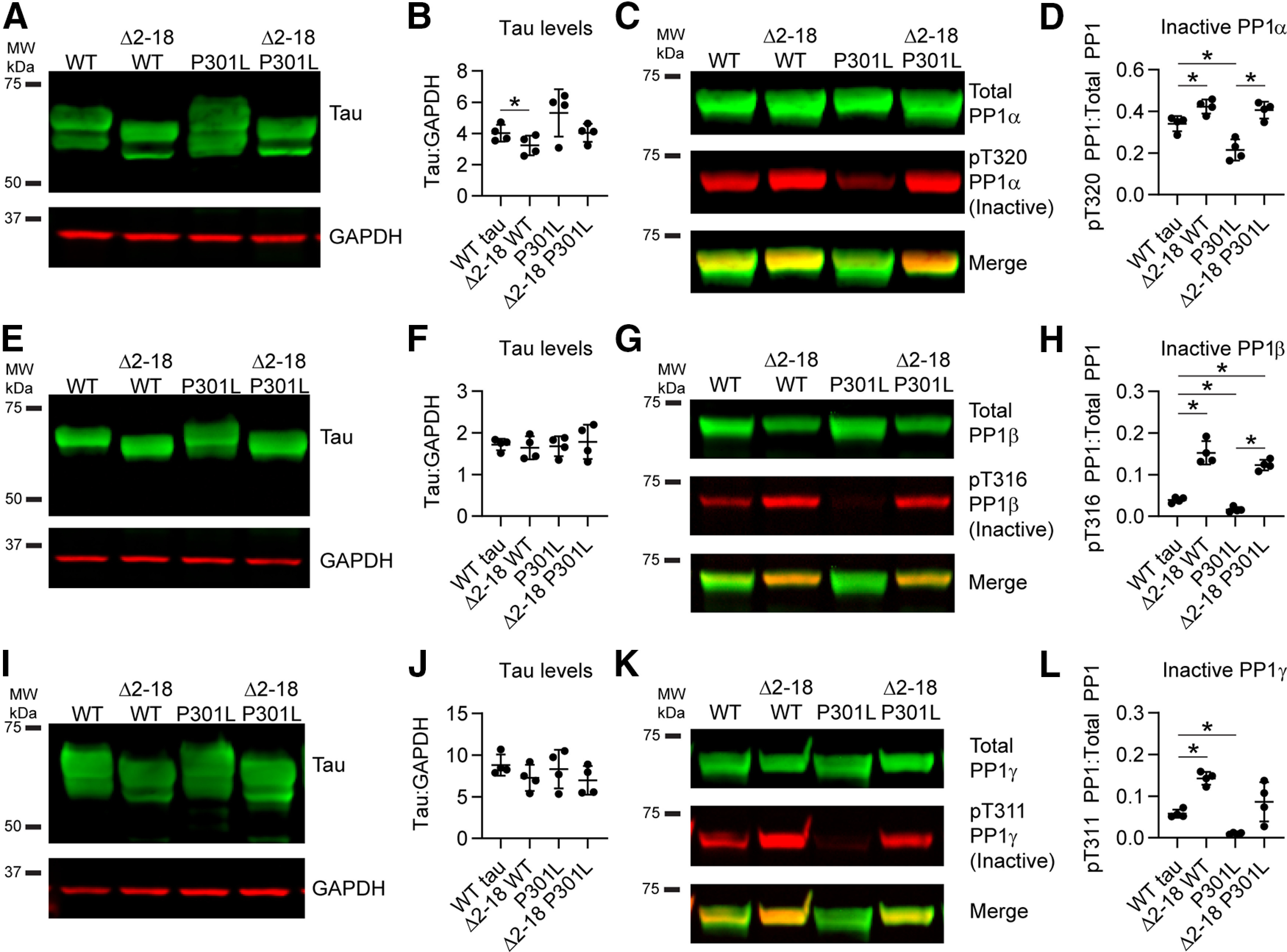
Tau-induced increases in active PP1 is dependent on PAD. PAD, a conformationally displayed biologically active motif in the extreme amino terminus of tau, was deleted in WT and P301L tau by removing amino acids 2–18 (Δ2–18). ***A***, ***B***, The full-length and Δ2–18 constructs were expressed in HEK293 cells, in addition to PP1α, at similar levels (***B***; WT tau, 4.02 ± 0.54; Δ2–18 WT, 3.24 ± 0.63; P301L, 5.32 ± 1.52; Δ2–18 P301L, 4.04 ± 0.58). ***C***, Levels of active PP1α (as indicated by changes in inactive pT320 PP1) were identified using a phospho-Thr320 PP1 antibody (red), and total levels were detected using an anti-HaloTag antibody (green). ***D***, Deletion of PAD resulted in significantly lower levels of active PP1 compared with expression of full-length tau in both WT and P301L backgrounds (WT tau, 0.3409 ± 0.0366; Δ2–18 WT, 0.4232 ± 0.0336; P301L, 0.2147 ± 0.0507; Δ2–18 P301L, 0.4062 ± 0.0406). ***E***, ***F***, WT tau, Δ2–18 WT, P301L, and Δ2–18 P301L were expressed at similar levels (***F***) along with PP1β (WT tau, 1.71 ± 0.14; Δ2–18 WT, 1.64 ± 0.28; P301L, 1.68 ± 0.24; Δ2–18 P301L, 1.78 ± 0.41). ***G***, Immunoblotting of total PP1 (green) and inactive PP1β (red, pT320 PP1 antibody corresponds to pT316 in PP1β). ***H***, Active PP1β levels decreased in the presence of Δ2–18 WT and Δ2–18 P301L compared with full-length WT and P301L tau (WT tau, 0.0393 ± 0.0067; Δ2–18 WT, 0.1525 ± 0.0281; P301L, 0.0162 ± 0.0062; Δ2–18 P301L, 0.1231 ± 0.0125). ***I***, WT tau, Δ2–18 WT, P301L, and Δ2–18 P301L in cells coexpressing PP1γ. ***J***, Quantification indicated similar levels of tau expression with all constructs (WT tau, 8.82 ± 1.27; Δ2–18 WT, 7.26 ± 1.57; P301L, 8.32 ± 2.32; Δ2–18 P301L, 6.96 ± 1.73). ***K***, Immunoblots of total PP1 (green) and inactive PP1γ (red, pT320 PP1 antibody corresponds to pT311 in PP1γ). ***L***, The Δ2–18 WT resulted in decreased levels of active PP1γ compared with full-length counterparts (WT tau, 0.0578 ± 0.0099; Δ2–18 WT, 0.1428 ± 0.0154; P301L, 0.0101 ± 0.0022; Δ2–18 P301L, 0.0862 ± 0.0471). P301L tau expression led to higher levels of active PP1 compared with WT tau, and this effect was reduced about ninefold on PAD deletion in Δ2–18 P301L. Tau signal was normalized to GAPDH loading control (***B***, ***F***, ***J***) and pT320 PP1 signal was normalized to total PP1 signal (***D***, ***H***, ***L***). Data in legend are reported in ratios of the signal intensities in arbitrary units. All data in figure are mean ± SD and were compared using repeated measures one-way ANOVAs with a Geisser–Greenhouse correction and Tukey's *post hoc* multiple comparison test, *p ≤ 0.05).

**Figure 10.**
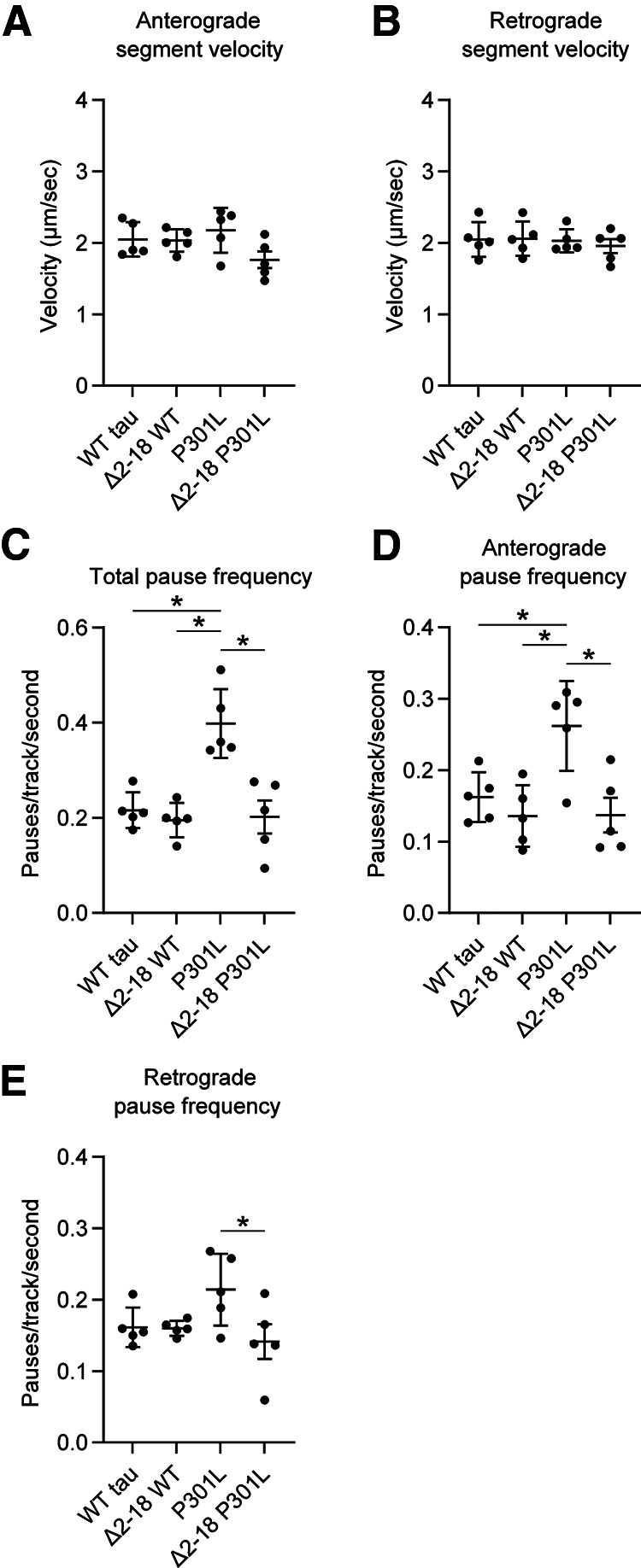
PAD deletion rescues P301L tau-induced increases in pause frequency. Transport of fluorescent synaptophysin was analyzed in the presence of full-length and PAD deletion (Δ2–18) WT and P301L tau constructs. ***A***, Anterograde segment velocity was not significantly altered (WT tau, 2.05 ± 0.24 µm/s; Δ2–18 WT, 2.03 ± 0.16 µm/s; P301L, 2.18 ± 0.31 µm/s; Δ2–18 P301L, 1.76 ± 0.26 µm/s). ***B***, No significant changes were detected in retrograde segment velocity (WT tau, 2.05 ± 0.24 µm/s; Δ2–18 WT, 2.06 ± 0.24 µm/s; P301L, 2.03 ± 0.16 µm/s; Δ2–18 P301L, 1.96 ± 0.22 µm/s). ***C***, The P301L tau induced a significant increase in total pause frequency compared with WT tau that was rescued by PAD deletion, whereas deletion of PAD in WT tau did not alter pause frequency (WT tau, 0.2158 ± 0.0375 pauses/s; Δ2–18 WT, 0.1951 ± 0.0363 pauses/s; P301L, 0.3980 ± 0.0723 pauses/s; Δ2–18 P301L, 0.2018 ± 0.0772 pauses/s). ***D***, The directionality of these effects was determined by separating pauses into the anterograde and retrograde directions. P301L again induced an increase in anterograde pause frequency that was rescued by PAD deletion in Δ2–18 P301L (WT tau, 0.1623 ± 0.0348 pauses/s; Δ2–18 WT, 0.1358 ± 0.0431 pauses/s; P301L, 0.2618 ± 0.0629 pauses/s; Δ2–18 P301L, 0.1370 ± 0.0540 pauses/s). ***E***, A significant change in retrograde pause frequency was not observed between WT tau and P301L tau. However, retrograde pause frequency with Δ2–18 P301L expression was significantly reduced compared with full-length P301L (WT tau, 0.1614 ± 0.0274 pauses/s; Δ2–18 WT, 0.1601 ± 0.0105 pauses/s; P301L, 0.2142 ± 0.0502 pauses/s; Δ2–18 P301L, 0.1415 ± 0.05,443 pauses/s). Data were compared using a one-way ANOVA with Tukey's multiple comparison test, and each data point represents an independent replicate (*n* = 5; the data are mean ± SD; *p ≤ 0.05).

Statistical analysis was performed using GraphPad Prism 9. Comparisons within the tau groups were made using one-way ANOVAs with Tukey's *post hoc* multiple comparison test (see Figs. 1, 6, 7*A*,*B*, 10). Repeated measures one-way ANOVAs with the Geisser–Greenhouse correction to account for violations of the assumptions of sphericity (as indicated here by an ε value <0.5) and Tukey's multiple comparison test were used for the cell lysate immunoblots (see Figs. 5, 9). A repeated measures one-way ANOVA with an assumption of sphericity and Tukey's multiple comparison test were used to analyze the PLA quantitation (see Figs. 4, 8). A repeated measures two-way ANOVA with an assumption of sphericity and Tukey's multiple comparison test was used for the pull-down assay (see Fig. 2, tau groups and PP1/Halo groups). Repeated measures ANOVAs were used to account for matching within datasets (e.g., cell culture runs). Two-way ANOVAS with Tukey's multiple comparison test were used for comparisons in the nBRET assay (see Fig. 3, tau groups and PP1/Halo groups) and axonal transport experiments with PP1 knockdown (see Fig. 8, tau groups and shRNA groups). The *p* values reported from Tukey's *post hoc* test within each ANOVA are adjusted for multiple comparisons. Significance was established at *p* ≤ 0.05. All experiments were performed three to six independent times, and replicates are described in each figure legend and in Materials and Methods. The horizontal lines and error bars represent mean ± SD.

## Results

### FTLD mutant tau impairs axonal transport in the squid axoplasm model

Pathogenic forms of tau, including WT aggregates and several disease-related monomeric forms of the protein, disrupted FAT in a squid axoplasm model (Kanaan et al., 2011). This effect was associated with changes in tau conformation that lead to aberrant exposure of PAD in the amino terminus and activation of a signaling pathway downstream of PP1 that has a negative impact on kinesin-1-based anterograde FAT (LaPointe et al., 2009; Kanaan et al., 2011; Cox et al., 2016; Tiernan et al., 2016).

Using vesicle motility assays in isolated squid axoplasm, we first tested whether monomeric P301L or R5L mutant tau affects FAT. Following perfusion of 2 μm monomeric WT tau, a near-physiological concentration (Binder et al., 1985; Kanaan and Grabinski, 2021), FAT rates remained unchanged for 50 min (Fig. 1*A*), as previously reported (Morfini et al., 2007; LaPointe et al., 2009; Kanaan et al., 2011, 2012; Cox et al., 2016; Tiernan et al., 2016). In contrast, FAT rates decreased in both anterograde and retrograde directions on perfusion of axoplasms with 2 μm monomeric P301L tau (Fig. 1*B*) or R5L tau (Fig. 1*C*). We calculated the means of the rate readings over the last 20 min of the assay to identify any differences among the conditions. Perfusion with P301L or R5L mutant tau significantly impaired the rate of anterograde FAT when compared with WT tau (Fig. 1*D*; *F*_(2,10)_ = 25.25, *p* = 0.0001; P301L vs WT, *p* = 0.0082; R5L vs WT, *p* = 0.0001). P301L and R5L tau perfusion also reduced retrograde FAT rates compared with WT tau (Fig. 1*E*; *F*_(2,10)_ = 16.68, *p* = 0.0007; P301L vs WT, *p* = 0.0079; R5L vs WT, *p* = 0.0008). These data demonstrate that in the isolated axoplasm preparation, both the P301L and R5L mutations confer on monomeric tau a toxic effect on FAT.

### The interaction between tau and PP1 is enhanced by the P301L tau mutation

Next, we sought to evaluate whether tau interacts with any of the three major PP1 isoforms in mammalian nerve tissues, PP1α, PP1β, and PP1γ1 (da Cruz e Silva et al., 1995). We began by performing pull-down experiments using HEK293T cells cotransfected with plasmids encoding PP1-HaloTag (N terminal) or HaloTag proteins and WT, P301L, or R5L tau (C-terminal NanoLuciferase-tagged; Fig. 2*A*). Immunoblotting experiments showed no significant differences in tau or PP1 expression across the conditions (Fig. 2*B*,*D*,*F*, Input). All tau proteins eluted in the Halo-PP1α pull-down elution sample (Fig. 2*B*, red box, left), but not with HaloTag alone (Fig. 2*B*, red box, right). Quantitative data showed increased P301L tau levels in pull-down elution, compared with WT and R5L tau, indicating enhanced binding of mutant P301L tau to PP1α (Fig. 2*C*; Tau, *F*_(2,6)_ = 7.348, *p* = 0.0244; PP1α, *F*_(1,3)_ = 11.46, *p* = 0.0429; Interaction, *F*_(2,6)_ = 7.339, *p* = 0.0244; P301L vs WT tau, *p* = 0.0311; P301L vs R5L tau, *p* = 0.0198). We normalized the tau signal to the change in Halo signal from before to after pull down (Fig. 2*B*, bottom) because the HaloTag covalently binds the resin and does not elute in these conditions. In the Halo-PP1β pull-down samples, we detected trace amounts of tau (Fig. 2*D*), but the degree of interaction was about fivefold lower than with PP1α or PP1γ. The P301L tau mutation did not significantly increase the interaction with PP1β compared with WT tau (Fig. 2*E*; Tau, *F*_(2,6)_ = 5.093, *p* = 0.0509; PP1β, *F*_(1,3)_ = 11.51, *p* = 0.0427; Interaction, *F*_(2,6)_ = 5.765, *p* = 0.0401; P301L vs WT tau, *p* = 0.1020) but did compared with R5L tau (*p* = 0.0287). Finally, all three tau forms eluted with the pull downs of the Halo-PP1γ isoform (Fig. 2*F*). Again, P301L tau induced a significant increase in binding efficiency compared with WT and R5L tau (Fig. 2*G*; Tau, *F*_(2,6)_ = 17.28, *p* = 0.0032; PP1γ, *F*_(1,3)_ = 30.89, *p* = 0.0115; Interaction, *F*_(2,6)_ = 16.86, *p* = 0.0034; P301L vs WT tau, *p* = 0.0033; P301L vs R5L tau, *p* = 0.0008). Notably, the level of R5L pulled down with PP1α (*p* = 0.9962), PP1β (*p* = 0.8256), or PP1γ (*p* = 0.9999) was unchanged when compared with WT, suggesting that this mutation does not alter the extent of physical interaction between tau and PP1. We did not detect tau in any of the control samples in concurrent pull downs that were performed with HaloTag-only (i.e., identical conditions other than no PP1 expression), which effectively controls for nonspecific binding of HaloTag protein, nonspecific interactions with the Halo-Link resin, and other experimental conditions. Collectively, these data suggest that tau interacts with PP1α and PP1γ and that this interaction is significantly enhanced by the pathogenic P301L tau mutation but not the R5L mutation.

Results from pull-down experiments provide evidence supporting a protein–protein interaction between tau and selected PP1 isoforms. We used nBRET assays to further evaluate this interaction in living cells (Fig. 3*A*). This complementary approach avoids issues related to cell lysis before assessing the interaction. First, we performed a donor saturation assay by transfecting the cells with a constant concentration of transfected donor DNA and varied concentrations of acceptor DNA. A specific interaction is indicated by high-signal, hyperbolic curves that display saturation of the nBRET ratios when plotted against the DNA ratio (Mercier et al., 2002). The nBRET ratio curves produced from increasing Halo-PP1α with either WT, P301L, or R5L tau (with C-terminal Nano Luciferase tags) indicate specific interactions between PP1α and each of the tau proteins within cells (Fig. 3*B*). In contrast, nBRET ratios generated from cells expressing Halo-PP1β with any of the tau constructs did not support interaction between the proteins because of the overall low nBRET signal (Fig. 3*C*). Coexpression of Halo-PP1γ with tau produced curves indicating a specific interaction with each of the tau forms (Fig. 3*D*). The low level of interaction with PP1β compared with PP1α and PP1γ aligns well with data from co-pull-down experiments (Fig. 2). To control for potential nonspecific interactions between tau-NanoLuc and the HaloTag itself, we coexpressed the tau proteins with the HaloTag protein alone and found no evidence of specific interaction between any of the tau proteins and the HaloTag (Fig. 3*E*).

We repeated the nBRET assay in four independent experiments at a single DNA ratio of 100:1 to perform statistical comparisons among tau groups and among Halo-PP1 and the Halo-only control. The nBRET ratios with P301L tau and Halo-PP1α significantly increased compared with WT and R5L tau with Halo-PP1α and significantly increased for all tau groups compared with the Halo-only control groups (Fig. 3*F*; Tau, *F*_(2,18)_ = 58.78, *p* < 0.0001; Halo-PP1α, *F*_(1,18)_ = 273.8, *p* < 0.0001; Interaction, *F*_(2,18)_ = 47.98, *p* < 0.0001; P301L vs WT tau, *p* < 0.0001; P301L vs R5L tau, *p* < 0.0001; Halo-PP1α vs Halo-only WT tau, *p* = 0.0006; P301L tau, *p* < 0.0001; R5L tau, *p* = 0.0006). None of the individual comparisons with Halo-PP1β were statistically significant, again suggesting a weak or nonexistent interaction with this PP1 isoform (Fig. 3*G*; Tau, *F*_(2,18)_ = 2.381, *p* = 0.1209; Halo-PP1β, *F*_(1,18)_ = 6.149, *p* = 0.0233; Interaction, *F*_(2,18)_ = 0.8514, *p* = 0.4433). Similar to PP1α, the nBRET ratio of P301L tau and Halo-PP1γ significantly increased when compared with WT and R5L tau, and all three tau proteins significantly increased the ratio with Halo-PP1γ coexpression compared with Halo-only controls (Fig. 3*H*; Tau, *F*_(2,18)_ = 60.49, *p* < 0.0001; Halo-PP1γ, *F*_(1,18)_ = 225.7, *p* < 0.0001; Interaction, *F*_(2,18)_ = 50.36, *p* < 0.0001; P301L vs WT tau, *p* < 0.0001; P301L vs R5L tau, *p* < 0.0001; Halo-PP1γ vs Halo-only WT tau, *p* = 0.0009; P301L tau, *p* < 0.0001; R5L tau, *p* = 0.0079). The nBRET ratios for R5L were similar to those for WT with PP1α (*p* = 0.9610), PP1β (*p* = 0.9998) or PP1γ (*p* = 0.9739), confirming that this mutation does not alter the degree of interaction in an in-cell assay. Considered together, these data indicate that when coexpressed in living cells, tau interacts with PP1α and PP1γ but only very weakly or not at all with PP1β. Additionally, the P301L mutation robustly enhances the interaction with PP1α and PP1γ compared with WT tau, whereas the R5L mutation does not, which is consistent with results from experiments in Figure 2.

### Tau associates with endogenous PP1 in mammalian neurons

After using an immortalized cell line to identify mutation- and isoform-specific effects, we next wanted to identify interactions in neurons. Using lentiviruses, we expressed the various tau constructs in primary rat hippocampal neuron cultures, which typically produce low levels of overexpression. We used PLA to identify relative differences in the association between tau and PP1 in the neurons expressing WT, P301L, and R5L tau. The assay uses antibody pairs that selectively recognize putative interactor proteins. Secondary antibodies, linked to complementary oligonucleotide sequences, bind the primary antibodies. The complementary oligo sequences are ligated *in situ* if the primary antibody epitopes from the two proteins are near each other (<40 nm). Following amplification and hybridization with fluorescent oligonucleotides, ligated oligos are indicated by bright puncta (Fig. 4*A*, green).

We probed neurons with Tau7, a mouse monoclonal antibody that binds to the C terminus of tau (Horowitz et al., 2004) and a PP1 rabbit polyclonal antibody that recognizes all PP1 isoforms in an immunoblot (Fig. 4*B*). Quantification of the PLA signal indicated that the P301L mutation enhanced the association between tau and PP1 compared with WT and R5L tau (Fig. 4*C*; *F*_(2,4)_ = 42.97, *p* = 0.0020; P301L vs WT tau, *p* = 0.0021; P301L vs R5L, *p* = 0.0046). Neurons expressing WT human tau presented PLA puncta, indicating a tau-PP1 association in neurites and cell bodies (Fig. 4*D–F*). We identified increased concentrations of puncta in neurons expressing P301L tau that also localized to neurites and soma (Fig. 4*C*,4*G–I*). R5L-expressing neurons also displayed puncta with similar patterns of subcellular localization, but there was no increase compared with WT (Fig. 4*C*,*J–L*). The control conditions included primary antibody deletions of Tau7 (Fig. 4*M*), PP1 (Fig. 4*N*), and both antibodies (Fig. 4*O*), all of which showed little to no background signal in the neurons. Finally, we identified associations between endogenous rat tau and PP1 in nontransduced cells (Fig. 4*P–R*). These results strongly support an interaction between tau and PP1 in mammalian neurons under physiological conditions. WT tau and both mutants interact with PP1, but the P301L mutation increases the binding of PP1 to tau relative to WT, whereas the R5L mutation does not increase PP1 binding over WT tau.

### FTLD-tau mutations increase levels of active PP1

After establishing tau interactions with PP1α and PP1γ, we determined whether tau also affected levels of active PP1 in cells cotransfected with GFP, WT, P301L, or R5L tau. First, we confirmed that similar levels of tau expression, normalized to GAPDH levels, were present in the experiments with PP1α coexpression (Fig. 5*A*,*B*; *F*_(1.585,4.754)_ = 2.764, *p* = 0.1610). Next, we probed lysates by immunoblotting using an anti-pT320 PP1 antibody that recognizes the inhibitory phosphoT320 site in PP1α and analogous sites T316 in PP1β and T311 in PP1γ (Dohadwala et al., 1994; Hou et al., 2013), as well as a phosphorylation-independent anti-HaloTag antibody (Total PP1). P301L tau significantly increased the level of active PP1α (as indicated by reduced inactive pT320 PP1α signal normalized to total PP1α signal), compared with WT-tau-expressing cells (Fig. 5*C*,*D*; *F*_(1.410,4.229)_ = 4.705, *p* = 0.0885; P301L vs WT tau, *p* = 0.0031) but none of the other comparisons were statistically different (R5L vs WT tau, *p* = 0.1698). We confirmed similar levels of tau expression in the PP1β experiments (Fig. 5*E*,*F*; *F*_(1.095,3.286)_ = 0.9685, *p* = 0.4025). Expressing each of the mutant tau proteins led to a significant increase in the level of active PP1β when compared with GFP and WT tau (Fig. 5*G*,*H*; *F*_(1.693,5.080)_ = 41.45, *p* = 0.0008; P301L tau vs GFP, *p* = 0.0216; R5L tau vs GFP, *p* = 0.0029; P301L vs WT tau, *p* = 0.0236; R5L vs WT tau, 0.0211). We then expressed similar levels of tau with PP1γ (Fig. 5*I*,*J*; *F*_(1.113,3.338)_ = 1.128, *p* = 0.3696). Again, both P301L and R5L mutant tau proteins significantly increased the levels of active PP1γ compared with GFP and WT tau (Fig. 5*K*,*L*; *F*_(1.048,3.143)_ = 30.28, *p* = 0.0103; P301L tau vs GFP, *p* = 0.0262; R5L tau vs GFP, *p* = 0.0328; P301L vs WT tau, *p* = 0.0070; R5L vs WT tau, *p* < 0.0001). These results show that P301L or R5L mutant coexpression with PP1 reduced levels of T320 phosphorylation in PP1β and PP1γ isoforms compared with control conditions (i.e., GFP and WT tau), and P301L tau expression reduced it in PP1α compared with GFP expression. This suggests that these FTLD-tau mutants are associated with increased levels of active PP1 in HEK293T cells.

After identifying the effect of tau on active PP1 levels in the HEK293T cell line, we set out to measure these effects on endogenous PP1 in primary rat hippocampal neurons by individually expressing the three tau constructs or a GFP control via lentiviral transduction. We immunoblotted the neuron lysates to confirm equal levels of protein loading and tau expression (Fig. 5*M*,*N*; *F*_(1.270,3.810)_ = 3.013, *p* = 0.1632). Then we probed the lysates with the anti-pT320 PP1 antibody and a total PP1 antibody (Fig. 5*O*). Expression of P301L tau and R5L tau increased levels of active PP1 (indicated by decreased inactive pT320 PP1) compared with GFP-expressing neurons (Fig. 5*P*; *F*_(1.559,4.677)_ = 7.053, *p* = 0.0420; P301L tau vs GFP, *p* = 0.0403; R5L tau vs GFP, *p* = 0.0468). In contrast, WT tau expression did not significantly alter active PP1 levels (*p* = 0.9962). This supports an increase in active PP1 levels following expression of FTLD mutant tau, but not WT tau.

### FTLD mutant tau impairs axonal transport in mammalian neurons

Next, we tested whether our findings about the effects of tau on FAT in the squid axoplasm extended to mammalian cultured neurons. We cotransfected rat primary hippocampal neurons with mApple-synaptophysin (an established cargo protein of synaptic vesicle precursors) and either WT, P301L, or R5L mutant tau or a GFP control construct (Fig. 6*A*). Using live cell confocal microscopy to image vesicle transport along the axon (∼28 frames/s; Fig. 6*B*) we then measured transport characteristics via kymograph analysis (Fig. 6*C*). In GFP-expressing and all tau-expressing neurons, fluorescently tagged synaptic vesicle precursors displayed similar anterograde FAT rates (as indicated by the anterograde segment velocity; Fig. 6*D*; *F*_(3,19)_ = 1.369, *p* = 0.2824). When compared with GFP cells, we detected a small but significant increase in retrograde FAT rates (∼20%) in neurons expressing each of the tau proteins (Fig. 6*E*; *F*_(3,19)_ = 5.900, *p* = 0.0051; WT tau vs GFP, 0.0353; P301L tau vs GFP, *p* = 0.0103; R5L tau vs GFP, 0.0104). We also analyzed the impact of tau expression on the pause frequency of fluorescently labeled vesicles, as PP1 activation by pathogenic tau promotes detachment of kinesin-1 from its transported organelle cargoes (Morfini et al., 2002; Kanaan et al., 2011). Compared with GFP control, WT tau did not significantly alter cargo pause frequency (Fig. 6*F*; *F*_(3,19)_ = 32.58, *p* < 0.0001; WT tau vs GFP: *p* = 0.9034). In contrast, P301L tau expression significantly increased the frequency of cargo pauses compared with both GFP (*p* < 0.0001) and WT tau (*p* < 0.0001, as did R5L tau expression compared with both GFP and WT tau (both *p* < 0.0001). To evaluate directionality-related effects on FAT, we further separated total pause frequency data for cargo traveling in the anterograde or retrograde directions. Expression of P301L tau or R5L tau significantly increased anterograde pause frequency compared with both GFP- and WT tau-expressing neurons (Fig. 6*G*; *F*_(3,19)_ = 5.990, *p* = 0.0047; P301L vs GFP, 0.0471; P301L vs WT tau, *p* = 0.0345; R5L tau vs GFP, *p* = 0.0341; R5L tau vs WT tau, *p* = 0.0252). Retrograde pause frequency also increased with expression of P301L tau compared with GFP (*p* = 0.0064) and WT tau (*p* = 0.0178; Fig. 6*H*; *F*_(3,19)_ = 8.853, *p* = 0.0007) and R5L tau induced the same effects (R5L tau vs GFP, *p* = 0.0045; R5L tau vs WT tau, *p* = 0.0120). These data suggest that although WT tau expression does not have an impact on cargo pausing, mutant P301L tau and R5L tau expression significantly increase cargo pause frequency. On the other hand, FAT rates were unaltered (anterograde FAT) or slightly increased (retrograde FAT) by all three forms of tau compared with control conditions.

### FTLD mutant tau impairs FAT via a PP1γ-dependent mechanism in hippocampal neurons

Next, we determined whether the P301L tau-induced effect on FAT documented in Figure 6 was dependent on PP1. We designed PP1 isoform-specific shRNAs to knock down expression of either PP1α or PP1γ because those isoforms displayed a clear interaction and activation with tau in our previous assays. Isoform-specific knockdown provides specificity while helping avoid toxic effects elicited by pharmacological PP1 inhibitors that target all isoforms. To confirm effectiveness of each shRNA, we measured shRNA-mediated knockdown of a *Renilla* luciferase-PP1 isoform fusion construct in a dual luciferase assay. The PP1α shRNA produced 87% knockdown when compared with controls expressing the reporter. The control nontargeting shRNA did not significantly alter expression when compared with the empty controls (i.e., 103% of control; Fig. 7*A*). The PP1γ shRNA produced 90% knockdown, whereas the signal with control shRNA was 102% of empty vector controls (Fig. 7*B*). To confirm knockdown in rat primary neurons we transduced them with lentiviruses expressing PP1α-shRNA, PP1γ-shRNA, or control shRNA. Using ddPCR we determined the mRNA concentrations from cell lysates under each condition, then normalized them to *rplp0* reference gene concentrations. The ddPCR analysis showed that PP1α mRNA levels were reduced 71% by PP1α-specific shRNA and only 19% by PP1γ-specific shRNA compared with the control shRNA (Fig. 7*C*). Similarly, PP1γ mRNA levels were reduced 70% by PP1γ-specific shRNA expression and only 9% by PP1α-specific shRNA when compared with the control shRNA treatment (Fig. 7*D*). These data validate that each PP1 isoform-specific shRNA produces target knockdown with low off-target (i.e., other PP1 isoform) effects in cultured hippocampal neurons.

Having established the knockdown specificity and effectiveness of shRNAs, we performed triple transfections of primary rat hippocampal neurons aimed at coexpressing (1) shRNA targeting PP1α or PP1γ or the nontargeting control shRNA; (2) WT, P301L, or R5L tau; and (3) mApple-synaptophysin in the various combinations. The PP1 isoform-specific shRNAs did not significantly affect anterograde transport velocity (Fig. 7*E*; Tau, *F*_(2,45)_ = 1.128, *p* = 0.3325; shRNA, *F*_(2,45)_ = 5.053, *p* = 0.0105; Interaction, *F*_(4,45)_ = 0.1333, *p* = 0.9693). R5L tau produced a small but significant increase in retrograde FAT compared with WT tau in the control shRNA condition (Fig. 7*F*; Tau, *F*_(2,45)_ = 1.999, *p* = 0.1473; shRNA, *F*_(2,45)_ = 3.788, *p* = 0.0302; Interaction, *F*_(4,45)_ = 1.148, *p* = 0.3464; R5L vs WT tau, *p* = 0.0333). These data indicate that exogenous tau expression did not affect FAT rates significantly, regardless of shRNA treatment groups.

We next examined whether PP1 shRNAs abolished the mutant-tau-induced effects on cargo pausing frequency. As expected from results in Figure 6, expressing P301L tau or R5L tau in the presence of control shRNA led to significantly increased total pause frequency compared with neurons expressing WT tau and control shRNA (Fig. 7*G*; Tables 1, 2; Tau, *F*_(2,45)_ = 17.61, *p* < 0.0001; shRNA, *F*_(2,45)_ = 16.61, *p* < 0.0001; Interaction, *F*_(4,45)_ = 4.463, *p* = 0.0040). Compared with expression of WT tau, the PP1α shRNA did not significantly modify the increase in total pause frequency induced by mutant tau expression (Fig. 7*G*). In contrast, PP1γ shRNA significantly reduced the total pause frequency in P301L- and R5L-expressing neurons to levels observed in WT neurons with control shRNA (Fig. 7*G*, dotted line). These data indicate that the increase in FAT pause frequency induced by P301L or R5L tau expression is eliminated by specific knockdown of PP1γ, but not PP1α.

To further investigate the specificity of mutant tau effects on cargo pausing and the relationship of these effects to PP1γ knockdown, pause frequencies were binned for cargo traveling in the anterograde or retrograde FAT directions. Here, we found that PP1γ knockdown rescued mutant-tau-induced pausing defects in the anterograde direction. As observed with no shRNA treatments (Fig. 6*G–I*), expression of P301L tau significantly increased anterograde pause frequency (Fig. 7*H*; Tables 1, 2; Tau, *F*_(2,45)_ = 15.80, *p* < 0.0001; shRNA, *F*_(2,45)_ = 20.94, *p* < 0.0001; Interaction, *F*_(4,45)_ = 2.133, *p* = 0.0923) compared with cells expressing WT tau with control shRNA treatment. Knockdown of PP1α did not alter this effect. In contrast, treating the P301L- or R5L-expressing neurons with PP1γ-shRNA eliminated the increased anterograde pause frequency, restoring pausing to WT tau levels (Fig. 7*H*).

We detected similar effects of tau expression on retrograde pause frequency, albeit lower in magnitude (Fig. 7*I*; Tables 1, 2; Tau, *F*_(2,45)_ = 5.193, *p* = 0.0093; shRNA, *F*_(2,45)_ = 9.244, *p* = 0.0004; Interaction, *F*_(4,45)_ = 3.921, *p* = 0.0082). As expected, P301L or R5L expression increased retrograde cargo pause frequency with control shRNA coexpression. We did not observe significant differences among any of the tau groups with knockdown of PP1α, likely because of the nonsignificant elevation in pause frequency noted with PP1α shRNA (observed with total, anterograde, and retrograde pausing). Again, PP1γ shRNA completely prevented the increase in retrograde pause frequency in P301L- and R5L-expressing neurons. Collectively, these data indicate that P301L and R5L tau produce pause frequency abnormalities in both directions and that specifically knocking down PP1γ robustly alleviates these abnormalities.

### P301L tau increases association with the PP1γ isoform in primary neurons

We repeated the PLA experiments with an antibody that specifically recognizes the PP1γ isoform to confirm an association between it and tau as well as to determine how FTLD mutations alter the association (Fig. 8*A*). Expression of P301L tau in the neurons increased PLA puncta compared with WT tau expression (Fig. 8*B*; *F*_(2,4)_ = 8.785, *p* = 0.0344; P301L vs WT tau, *p* = 0.0326). No significant differences existed between WT tau and R5L tau (*p* = 0.5210). Primary deletion of the various antibodies resulted in little to no background signal (Figs. 4*N*,*O*, 8*C*). WT tau-expressing neurons displayed PLA puncta (Fig. 8*D*), present in the neuronal processes (Fig. 8*E*) and cell bodies (Fig. 8*F*) indicating an association with the PP1γ isoform specifically. Expression of mutant P301L tau increased the number of puncta compared with WT tau expression (Fig. 8*G–I*). R5L tau also interacted with PP1γ in the neurons at similar levels to WT tau (Fig. 8*J–L*). Notably, these findings mirror the neuron PLA data using a pan-PP1 antibody (Fig. 4) and the data from HEK293T cells (Figs. 2, 3). We also identified associations between endogenous rat tau and PP1γ in untransduced neurons (Fig. 8*M–O*). Together, this supports a neuronal association between tau and the PP1γ isoform, found to be the mediator of the mutant-tau-induced effects on FAT (Fig. 7).

### FTLD mutant tau impairs FAT via a PAD-dependent mechanism in hippocampal neurons

Previously, the PAD (amino acids 2–18 in tau) was identified as necessary and sufficient to induce PP1-dependent transport defects in the squid axoplasm (LaPointe et al., 2009; Kanaan et al., 2011). Disease-relevant tau modifications are associated with conformation changes in this region that are early markers of tau dysfunction in tauopathies (Kanaan et al., 2011; Combs et al., 2016; Combs and Kanaan, 2017). We first sought to determine the relevance of PAD to the activation of PP1 by deleting amino acids 2–18 in both WT and P301L tau then expressing them with individual PP1 isoforms. We confirmed similar levels of tau expression, normalized to GAPDH loading control, for experiments with PP1α (Fig. 9*A*,*B*; *F*_(1.141,3.423_ = 4.431, *p* = 0.1146; WT tau vs Δ2–18 WT tau, *p* = 0.0113), PP1β (Fig. 9*E*,*F*; *F*_(1.066,3.197_ = 0.2118, *p* = 0.6896), and PP1γ (Fig. 9*I*,*J*; *F*_(1.287,3.860_ = 1.269, *p* = 0.3455). Expressing Δ2–18 WT tau and Δ2–18 P301L tau resulted in reduced active PP1α (i.e., higher inactive PP1α to total PP1) compared with full-length counterparts (Fig. 9*C*,*D*; *F*_(1.532,4.595_ = 27.27, *p* = 0.0033; WT tau vs Δ2–18 WT tau, *p* = 0.0018; P301L tau vs Δ2–18 P301L tau, *p* = 0.0463). Importantly, PAD deletion rescued the P301L-induced change from WT tau (WT tau vs P301L, *p* = 0.0276; Δ2–18 P301L tau vs WT tau, *p* = 0.2834). Similarly, P301L tau resulted in an increase in active PP1β compared with WT tau (*p* = 0.0232), and deletion of PAD reduced active PP1β for both WT and P301L tau (Fig. 9*G*,*H*; *F*_(1.301,3.904_ = 106.0, *p* = 0.0005; WT tau vs Δ2–18 WT tau, *p* = 0.0113; P301L tau vs Δ2–18 P301L tau, *p* = 0.005). Finally, we detected lower levels of active PP1γ with Δ2–18 WT tau compared with full-length tau expression (Fig. 9*K*,*L*; *F*_(1.034,3.102_ = 19.12, *p* = 0.0205; WT tau vs Δ2–18 WT tau, *p* = 0.0196). P301L tau induced a significant increase in active PP1 compared with WT tau (*p* = 0.0092), and PAD deletion in a P301L background caused a ninefold reduction in active PP1γ that did not reach statistical significance (*p* = 0.1531) and a return to levels that were not significantly different from WT tau (*p* = 0.7579). These indicate that generally the PAD region in the N terminus of tau mediates its effects on increasing levels of active PP1.

Finally, we examined the role of PAD on the alterations in FAT of cargoes containing mApple-synaptophysin in primary rat neurons. We individually coexpressed full-length and Δ2–18 versions of WT and P301L tau with the synaptophysin construct then imaged and analyzed transport as above. Deletion of amino acids 2–18 did not influence anterograde (Fig. 10*A*; *F*_(3,16)_ = 2.431, *p* = 0.1029) or retrograde (Fig. 10*B*; *F*_(3,16)_ = 0.2275, *p* = 0.8758) velocities. Again, P301L tau increased total pause frequency compared with WT tau (Fig. 10*C*; *F*_(3,16)_ = 13.59, *p* = 0.0001; WT tau vs P301L tau, *p* = 0.0009) and PAD deletion rescued pause frequency to control levels (WT tau vs Δ2–18 P301L tau, *p* = 0.9815; P301L tau vs Δ2–18 P301L tau, *p* = 0.0004). We observed a similar effect on pause frequency in the anterograde direction whereby PAD deletion rescued the P301L-induced increases (Fig. 10*D*; *F*_(3,16)_ = 7.150, *p* = 0.0029; WT tau vs P301L tau, *p* = 0.0281; WT tau vs Δ2–18 P301L tau, *p* = 0.8536; P301L tau vs Δ2–18 P301L tau, *p* = 0.0056). We did not detect significant increases in retrograde pause frequency in this experiment (Fig. 10*E*; *F*_(3,16)_ = 3.080, *p* = 0.0574; WT tau vs P301L tau, *p* = 0.1963) but deletion of amino acids 2–18 in P301L tau significantly reduced pause frequency compared with full-length P301L tau expression (*p* = 0.0479). This indicates that alterations in FAT elicited by P301L are PAD dependent, as previously shown for other pathogenic forms of tau in the isolated squid axoplasm model (Kanaan et al., 2011).

## Discussion

Impairments in FAT are widely regarded as a major pathogenic event contributing to dying-back degeneration of neurons in AD and related tauopathies and are linked to pathogenic tau forms (Kneynsberg et al., 2017). Transgenic P301L tau mice, for example, recapitulate many aspects of tau toxicity including axonal degeneration (Lewis et al., 2000; Ludvigson et al., 2011; de Calignon et al., 2012). These studies also documented FAT impairments (Bertrand et al., 2013) before overt degeneration (Gilley et al., 2012; Majid et al., 2014) but did not examine underlying molecular mechanisms. Using two tau mutations that cause inherited tauopathies we provide novel mechanistic insights into how tau impairs FAT.

Tau-induced FAT impairments in the isolated squid axoplasm model are PAD dependent, and pharmacological PP1 inhibitors rescue the effects in this model system (LaPointe et al., 2009; Kanaan et al., 2011). This led us to examine whether FTLD mutant tau inhibited FAT in squid axoplasms and in mammalian hippocampal neurons and to identify specific PP1 isoforms mediating the potential toxic effect. Unlike most other pathogenic tau forms that impair anterograde FAT, P301L and R5L tau disrupted both anterograde and retrograde FAT in the squid axoplasm and primary rat hippocampal neurons. Based on our findings, the bulk of this disruption occurred through a specific PAD- and PP1γ-dependent mechanism. The negative impact on retrograde FAT is similar to that of phosphomimics at select sites in tau (e.g., S422, S199, and S202), which we previously tested in isolated squid axoplasm (Tiernan et al., 2016; Morris et al., 2020), suggesting that some pathogenic tau forms may also disrupt pathways involved in the regulation of retrograde FAT and cellular processes dependent on this direction of transport (e.g., neurotrophic factor signaling).

We detected FAT dysfunction through distinct readouts in the two assays. Because of the unique method of microscopy used to evaluate FAT in isolated axoplasm, effector proteins that promote kinesin release from either cargo (Morfini et al., 2004) or microtubules (Morfini et al., 2009, 2013) promote a reduction in FAT rate readout (Song et al., 2016). In primary neuron cultures, fluorescence-based confocal microscopic analysis of FAT allows separate evaluation of translocation rates from pauses. Our findings align with others showing that P301L tau does not impair FAT instantaneous velocity. For example, there was no apparent deficit in mitochondrial transport velocity in primary neurons or tibial nerve explants from P301L knock-in mice (pauses were not assessed; Gilley et al., 2012; Rodríguez-Martín et al., 2016). Collectively, the data reported here and previously indicate that pathologic forms of tau that cause disease in humans can significantly disrupt FAT in neurons.

PP1 involvement in tau-mediated FAT dysfunction is supported by multiple protein–protein interaction assays showing P301L tau robustly increased interaction with PP1. PP1 has more than 200 binding partners that modulate localization of PP1, enzymatic activity, degradation, and/or substrate recognition either directly or by preventing interaction with other regulators (Bollen et al., 2010; Peti et al., 2013), and the roles of individual PP1 isoforms are incompletely understood (Heroes et al., 2013). The only prior study addressing tau–PP1 interactions found that tau localized PP1 to microtubules (Liao et al., 1998). Several questions remained unanswered, including the specific PP1 isoforms involved and functional consequences of the interactions. Together, our work and that from Liao et al. (1998) demonstrate that tau interacts with and activates PP1, consistent with our working hypothesis that tau regulates signaling pathways involved in numerous cellular processes, including those involved in FAT modulation (Mueller et al., 2021).

Like tau, several known PP1-interacting partners contain low complexity domains, which are thought to impart flexibility and allow simultaneous interaction with multiple binding motifs in PP1 (Mukrasch et al., 2009; Marsh et al., 2010; Ragusa et al., 2010; Choy et al., 2012). The P301L mutation is located within the microtubule binding domain (MTBR) of tau and changes the ^301^PGGG motif immediately preceding the ^306^VQIVYK sequence involved in tau aggregation. Relatively little is known about specific global conformational effects elicited by the P301L mutation, but it seems to open conformation of tau in a microtubule-bound state while also reducing microtubule-binding affinity (Hasegawa et al., 1998; Dayanandan et al., 1999; Di Primio et al., 2017). Such conformation could expose the N-terminal PAD or the MTBRs to facilitate tau–PP1 binding and subsequent changes to PP1 activity. In contrast, the R5L mutation (within PAD) did lead to increased active PP1 levels without altering binding to PP1. These data suggest a potential two-component process by which tau acts as a scaffold protein, with the MTBR mediating the interaction between tau and PP1, whereas PAD is more directly involved in modulating PP1 activation. Supporting this, PAD deletion reduced levels of active PP1 in WT and P301L backgrounds and rescued P301L-dependent FAT effects in neurons, aligning with previous results from squid axoplasms (LaPointe et al., 2009; Kanaan et al., 2011). The specific impact of the R5L mutation on tau conformation is unknown, but it could induce alterations similar to other mutations that increase PAD exposure (Kanaan et al., 2011; Combs et al., 2016). Continued investigations into how specific tau regions functionally interact with various PP1 binding motifs and the basis for the specificity of interactions with PP1 isoforms are warranted.

PP1α, PP1β, and PP1γ are the main catalytic isoforms in brain and differ somewhat in expression, localization, and specificity for binding partners and substrates. Each PP1 isoform is found in mammalian axons and axon terminals, presumably in the appropriate position to regulate cargo delivery (Strack et al., 1999; Bordelon et al., 2005). PLA signal localized to neuronal projections, but additional experiments are needed to definitively identify subcellular differences. In the *in vitro* assays, we established that tau interacted similarly with PP1α and PP1γ, whereas much less interaction occurred with PP1β. PP1α and PP1γ share some regions with higher sequence homology than corresponding regions in PP1β (Scotto-Lavino et al., 2010). Some proteins, like neurabins, also interact with PP1α/γ but weakly with PP1β, helping localize PP1 to synapses and altering activity (Terry-Lorenzo et al., 2002). This specificity depends on multiple domains surrounding canonical PP1-binding sequences (Carmody et al., 2004), reinforcing the complexity of PP1 regulatory interactions. The agreement among our results in these assays, the neuronal PLA experiments, and those by Liao et al. (1998) using brain-purified microtubule fractions provides confidence that a tau–PP1 interaction is biologically relevant. Although mutant tau interacted similarly with PP1α or PP1γ in non-neuronal assays, our findings in mammalian neurons suggest that the tau–PP1γ interaction is specifically responsible for the main effects on FAT in hippocampal neurons. *In vivo*, the molecular basis of PP1 isoform-specific effects in neurons likely involves differences in subcellular localization or cell-type-specific expression of selected PP1 isoforms, as well as the binding partners.

Although tau is a known substrate for PP1 (Liu et al., 2005; Rahman et al., 2005) this does not preclude a PP1-regulatory function, and several other PP1-regulating proteins serve this dual role (Bollen et al., 2010). Direct regulation of neuronal PP1 activity also occurs through an inhibitory Cdk5-mediated phosphorylation at a C-terminal threonine residue (T320/T316/T311 in PP1α/β/γ, respectively) that is rapidly autodephosphorylated by active PP1 (Dohadwala et al., 1994; Hou et al., 2013), thus providing a good inverse marker for the pool of active PP1 in cells. We previously showed Cdk5 inhibition also reduces FAT through activation of the PP1/GSK3β pathway (Morfini et al., 2004). Here, we provided evidence that tau expression increased levels of active PP1 in cultured cells. Our studies cannot rule out the possibility that yet unidentified additional PP1 or tau-binding partners are involved in this regulatory function. In our model GSK3 is activated on dephosphorylation by PP1 and responsible for phosphorylating kinesin light chain, inducing cargo release, though additional studies are needed to characterize the interaction of GSK3 with tau. Likely because of its multifunctional role in various cellular processes, GSK3β knockdown via shRNA was too disruptive to FAT to be useful in this context (data not shown).

Collectively, these experiments describe a specific PP1γ-dependent molecular mechanism by which P301L and R5L tau promote FAT impairments in mammalian neurons by increasing levels of active PP1. Collectively, the available data highlight a mechanism where different tau mutations similarly elicit toxic effects on FAT. Specifically, the P301L mutation in the MTBR increases PP1 binding and subsequently increases active PP1, whereas the R5L mutation in PAD enhances active PP1 without increased binding of PP1 relative to WT tau. Both mutations disrupt FAT in hippocampal neurons, evidenced by increased cargo pausing, an event that is PAD dependent and specifically mediated by PP1γ. This, and several other studies, now support our working model in which pathologic tau modifications (i.e., aggregation, abnormal phosphorylation, and FTLD mutations) alter PP1γ function resulting in dysregulation of FAT (Kanaan et al., 2013; Brady and Morfini, 2017). Given the large number of substrates targeted by PP1 isoforms, aberrant substrate activation may induce toxicity by disrupting the homeostasis of signaling pathways involved in the regulation of FAT and other cellular processes in tauopathies.
